# The Structural Determinants for α_1_-Adrenergic/Serotonin Receptors Activity among Phenylpiperazine-Hydantoin Derivatives

**DOI:** 10.3390/molecules26227025

**Published:** 2021-11-20

**Authors:** Katarzyna Kucwaj-Brysz, Anna Dela, Sabina Podlewska, Marek Bednarski, Agata Siwek, Grzegorz Satała, Kinga Czarnota, Jadwiga Handzlik, Katarzyna Kieć-Kononowicz

**Affiliations:** 1 Department of Technology and Biotechnology of Drugs, Faculty of Pharmacy, Jagiellonian University Medical College, 9 Medyczna Street, 30-688 Kraków, Poland; adela@pzh.gov.pl (A.D.); smusz@if-pan.krakow.pl (S.P.); kinga.czarnota@doctoral.uj.edu.pl (K.C.); j.handzlik@uj.edu.pl (J.H.); 2 Maj Institute of Pharmacology, Polish Academy of Sciences, 12 Smętna Street, 31-343 Kraków, Poland; satala@if-pan.krakow.pl; 3 Department of Pharmacodynamics, Faculty of Pharmacy, Jagiellonian University Medical College, Medyczna 9 Street, 30-688 Kraków, Poland; marek.bednarski@uj.edu.pl; 4 Department of Pharmacobiology, Faculty of Pharmacy, Jagiellonian University Medical College, Medyczna 9 Street, 30-688 Kraków, Poland; agat.siwek@uj.edu.pl

**Keywords:** adrenergic receptors, serotonin receptors, G protein-coupled receptors, selectivity, docking, molecular dynamics

## Abstract

Several studies confirmed the reciprocal interactions between adrenergic and serotoninergic systems and the influence of these phenomena on the pathogenesis of anxiety. Hence, searching for chemical agents with a multifunctional pharmacodynamic profile may bring highly effective therapy for CNS disorders. This study presents a deep structural insight into the hydantoin-arylpiperazine group and their serotonin/α-adrenergic activity. The newly synthesized compounds were tested in the radioligand binding assay and the intrinsic activity was evaluated for the selected derivatives. The computer-aided SAR analysis enabled us to answer questions about the influence of particular structural fragments on selective vs. multifunctional activity. As a result of the performed investigations, there were two leading structures: (a) compound **12** with multifunctional adrenergic-serotonin activity, which is a promising candidate to be an effective anxiolytic agent; (b) compound **14** with high α_1A_/α_1D_ affinity and selectivity towards α_1B_, which is recommended due to the elimination of probable cardiotoxic effect**.** The structural conclusions of this work provide significant support for future lead optimization in order to achieve the desired pharmacodynamic profile in searching for new CNS-modulating agents.

## 1. Introduction

**Figure 1:**
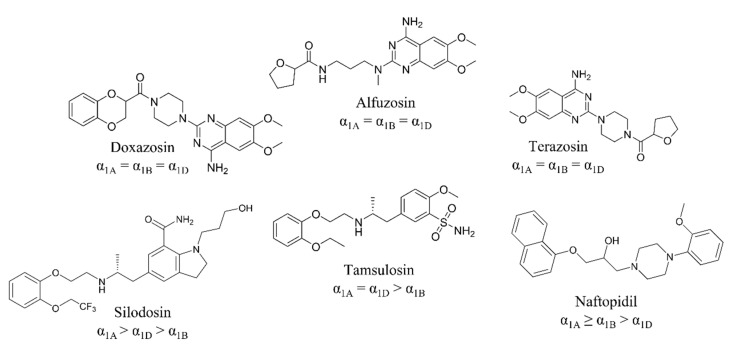
The α1-AR antagonists in clinical use and their sub-type α-AR (non)selectivity [3].

The α_1_-adrenergic receptors (ARs) belong to the great G-protein coupled receptor’s (GPCR’s) family and their role is the mediation of the sympathetic nervous system via binding endogenous catecholamines (adrenaline and noradrenaline) [1]. The deep characterization of tissue in the 1980s led to a division of ARs into three subtypes: α_1A_-AR, α_1B_-AR and α_1D_-AR [2]. Thanks to the ability to relax the smooth muscle in the prostate, the α_1_-AR blockers found mainly clinical use in the treatment of benign prostatic hyperplasia (BPH)—the enlargement of the prostate gland, which may further lead to lower urinary tract symptoms (LUTS), significantly decreasing quality of life [3]. The common side effect concerns hypotension, which is most probably due to the result of interactions with the subtype α_1B_-AR. For the tamsulosin—the selective α_1A_/α_1D_-AR antagonist (affinity to α_1A/_α_1D_ is 10-fold stronger than to α_1B_)—a significantly less undesired cardiovascular extension effect was observed than for the non-subtype selective α_1_-AR blocker named terazosin (Figure 1) [4].

Potential application of α_1_-AR blockers was indicated as an efficient treatment in cocaine use disorder (CUD) [5]. Additionally, the newest clinical studies confirmed that successful treatment with doxazosin is highly correlated with *ADRA1D* T-allele [6]. The very recent in vitro studies performed on novel derivatives of naftopidil indicate that α_1_-AR antagonists may be useful in prostate cancer therapy. The authors emphasized the significance of the subtype α_1_-AR selectivity for anticancer properties [7]. Finally, it is suggested that the regulation of α_1_-AR (α_1A_ and/or α_1B_) may play a neuroprotective role, being potentially useful for the treatment of neurological disorders (anxiety, depression, psychosis) [8]. For example, treatment with prazosin, an α_1_-AR inverse agonist, was successful in improving nightmare symptoms in patients with PTSD (posttraumatic stress disorder) [9]. Moreover, adrenergic receptors variants (including *ADRA1A*) were recently identified as the susceptibility factor for GAD (generalized anxiety disorders) [10].

**Figure 2:**
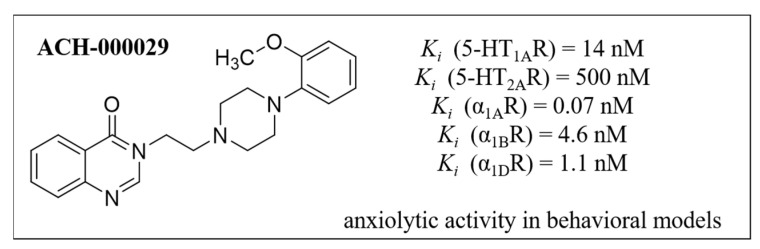
The chemical structure of ACH-000029 and its pharmacological profile.

Interestingly, several studies showed reciprocal interactions between adrenergic and serotoninergic systems and the influence of this phenomenon on the pathogenesis of anxiety [11,12]. The modulation of serotonin receptors, another protein belonging to the GPCR family, has already been indicated as a potential strategy for dealing with many CNS disorders including anxiety, especially receptors 5-HT_1A_ [13,14], 5-HT_2A_ [15] and 5-HT_7_ [16,17]. In this light, searching for multitargeted serotonin/adrenergic agents is a promising solution for the above-mentioned CNS dysfunction treatment. The proof of this hypothesis is confirmation of the anxiolytic activity of compound ACH-000029—a multifunctional quinazoline derivative—in animal models (Figure 2), thus highlighting the importance of further research in this field [12].

**Figure 3:**
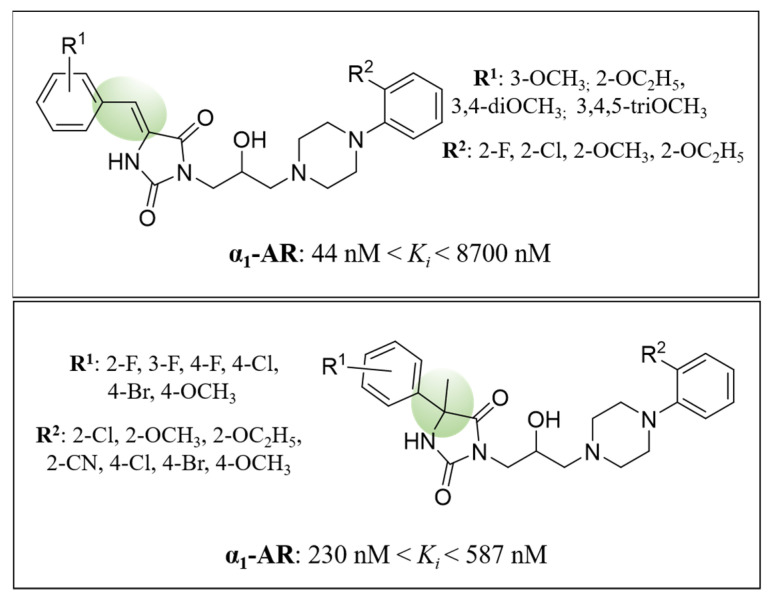
The structure and α_1_-AR affinities of the previously reported two groups of arylpiperazine derivatives of hydantoin.

Arylpiperazine is a very widely investigated chemical scaffold as it meets the main structural requirements of a pharmacophore model for many GPCR ligands (serotonin [18,19], histamine [20], adrenergic [21], dopamine receptors [22,23]). However, the non-pharmacophore moiety of the structure also maintains very important factors influencing the compound’s geometry and, therefore, its pharmacodynamic profile. The structure–activity relationship (SAR) analysis for previously reported arylpiperazine derivatives of 5-arylidene hydantoin with a 2-hydroxypropyl linker (Figure 3) led to the selection of the most beneficial substituent combinations, which provided high α_1_-AR affinity (for the best derivative: *K_i_* = 44.5 nM) [24]. Interestingly, another group of arylpiperazine hydantoins with 2-hydroxypropyl linker (Figure 3) shows lower α_1_-AR activity (*K*_i_ = 230 nM, for the best compound) but high and selective serotoninergic 5-HT_7_R activity (3 nM < *K_i_* < 94 nM), suggesting that the modulation of hydantoin substitution in position 5 (balancing between sp2 and sp3 hybridization of carbon atom C5) may be one of the main determinants for the serotoninergic/adrenergic affinity profile.

**Figure 4:**
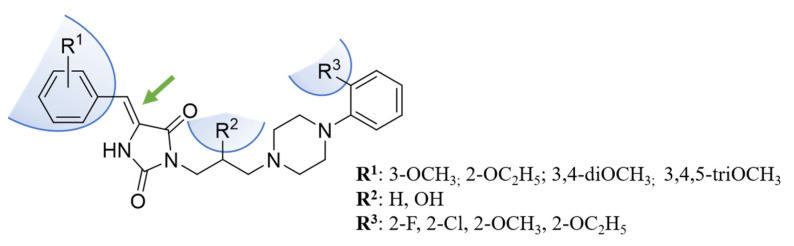
The herein investigated chemical modifications.

Hence, this study concerns the synthesis and in vitro pharmacological evaluation of novel 5-arylidenehydantoins (Figure 4) with very deep computer-aided insight into the structure–activity relationship in order to deal with the to-date unanswered questions, that is, (i) how the structural stiffening via double bond introduction influences the α_1_-AR and serotoninergic receptors’ activity; (ii) what the role of the hydroxy group in the linker is; (iii) what the best type of substitution of the benzylidene group is; (iv) what the key structural factors for α_1_-AR sub-type selectivity are.

All of the newly synthesized and previously reported derivatives by Czopek et al. (i.e., compound **14**) [25] were tested in radioligand binding assays to measure the affinity at α-adrenergic receptors, and the serotoninergic 5-HT_1A_R, 5-HT_6_R and 5-HT_7_R. Additionally, for four compounds with the highest activity at α-ARs (**10**, **12**, **14** and **16**), functional affinities at α-AR subtypes (α_1A_-AR in rat tail artery, at α_1B_-AR in mouse spleen and at α_1D_-AR in rat aorta) were determined. In order to perform a deep structural analysis, the crystallographic studies for representative compound **4** were reported. The molecular docking studies and dynamic simulations were elaborated to analyze interactions within protein-ligand complexes and their stability.

## 2. Results and Discussion

### 2.1. Chemical Synthesis

**Scheme 1:**
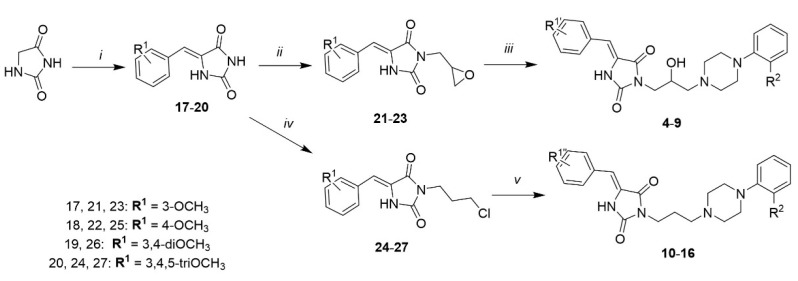
The synthetic pathway for compounds 4–16: (*i*) appropriate benzaldehyde, sodium acetate, acetic acid, reflux, 22–25 h, yield 36–72% (*ii*) 2,3-epoxypropan-1-ol, DEAD, PPh_3_, DMF 0 °C→rt, 2–3 h, yield 14–28%; (*iii*) phenylpiperazine derivative, microwave irradiation, 2–4 min, yield 19-57%; (*iv*) 3-chloropropan-1-ol, DEAD, PPh_3_, DMF 0 °C→rt, 2–3 h, yield 20–42%; (*v*) phenylpiperazine derivative, K_2_CO_3_, microwave irradiation, 2-4 min, yield 41–53%.

Compounds **4**–**16** were synthesized via a three-step chemical pathway according to Scheme 1. 5-Arylidene hydantoins (**17**–**20**) were obtained from hydantoin and a substituted benzaldehyde in Knoevenagel reaction. As we designed derivatives with two types of linkers (branched and unbranched), 2,3-epoxypropan-1-ol was used for N-substitution of position 3 of hydantoin in the Mitsunobu reaction, to obtain compounds **21**–**23** and 3-chloropropan-1-ol to give compounds **24**–**27**. The final step was a microwave-assisted condensation reaction. In the case of compounds with an epoxide group, no additional reagent was necessary, whereas for compounds with a chloroalkyl group, K_2_CO_3_ was used. 

### 2.2. Pharmacology

#### 2.2.1. Radioligand Binding Assays

The radioligand binding results showed that 11 out of the 14 tested compounds (**5**, **7**, **9**–**16**) bind to α-ARs with high affinity (*K_i_* < 100 nM). Compound **14** turned out to be the most active with *K_i_* = 11.9 nM. Interestingly, compounds **12** and **13** are strong dual 5-HT_1A_R/α-ARs ligands, whereas compound **5** indicates dual activity 5-HT_7_/α-AR (Table 1).

#### 2.2.2. Evaluation of Intrinsic Activity towards α_1_-AR Subtypes

For the representative compounds with the highest α_1_-AR affinity (**10**, **12**, **14** and **16**), intrinsic activity was measured (Table 2). All the tested compounds act as antagonists towards α_1A_-, α_1B_- and α_1D_-adrenergic receptors. However, compound **14** has significantly lower activity towards α_1B_ in comparison with other investigated derivatives. 

### 2.3. Cheminformatic Analysis and Molecular Modeling

#### 2.3.1. ChEMBL-Database-Oriented Searches for Structurally Similar Compounds

The analysis of the similarity coefficients of compounds present in the ChEMBL database [27] allowed us to evaluate their structural novelty. The threshold for the similarity coefficient Tanimoto (Tc) [28] applied was equal to 0.7. Excluding **2**, **3** and **14** (compounds previously published [25,26]), no structures within datasets corresponding to considered targets were found to be similar by more than 0.7 (in terms of Tc); which confirmed the high structural novelty of the presented compounds.

#### 2.3.2. Computer-Aided Structure-Activity Relationship towards Adrenergic Receptors

There were two main aims of molecular modeling studies carried out: explanation of the variation of activity of compounds **10**, **12**, **14** and **16** towards α_1_ subtypes (especially the significantly lower activity of compound **14** towards α_1B_), as well as an explanation of compound activity profiles towards serotonin receptors (in particular, the examination of the role of the -OH substituent and hybridization of the hydantoin C5).

**Figure 5:**
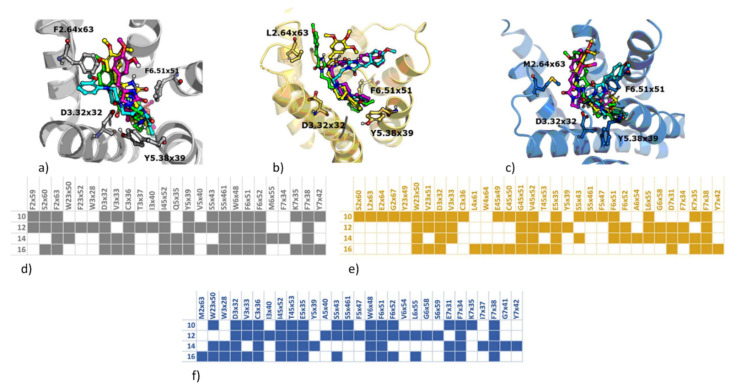
Docking results of compounds **10** (green), **12** (cyan), **14** (magenta), and **16** (yellow) to, (**a**) α_1A_R (docking poses), (**b**) α_1B_R (docking poses), (**c**) α_1D_R (docking poses), (**d**) interaction matrix with α_1A_R, (**e**) interaction matrix with α_1B_R, (**f**) interaction matrix with α_1D_R.

At first, the docking studies of **10**, **12**, **14** and **16** to α_1A_, α_1B_, and α_1D_ receptor models were carried out. They were performed with the use of the GPCRdb models [29] (inactive receptor states were used). The obtained docking poses for all the α_1_ receptor subtypes are presented in Figure 5.

The obtained docking results did not provide much insight into the observed variations in compound activity towards various α_1_ receptor subtypes. The IC_50_ values of **14** (in comparison with **10**, **12**, and **16**) are much higher for α_1A_ and α_1B_ (57.6 nM and 182 nM, respectively). However, for this receptor subtype, the conformation of **14** was similar to the orientations of other, more active compounds: for α_1A_, **14** adopted a similar binding pose to **16**_,_ for which the IC_50_ value was equal to 19.1 nM, whereas for α_1B_, the orientation of **14** was analogous to the binding pose of **12,** whose IC_50_ was equal to 8.5 nM.

**Figure 6:**
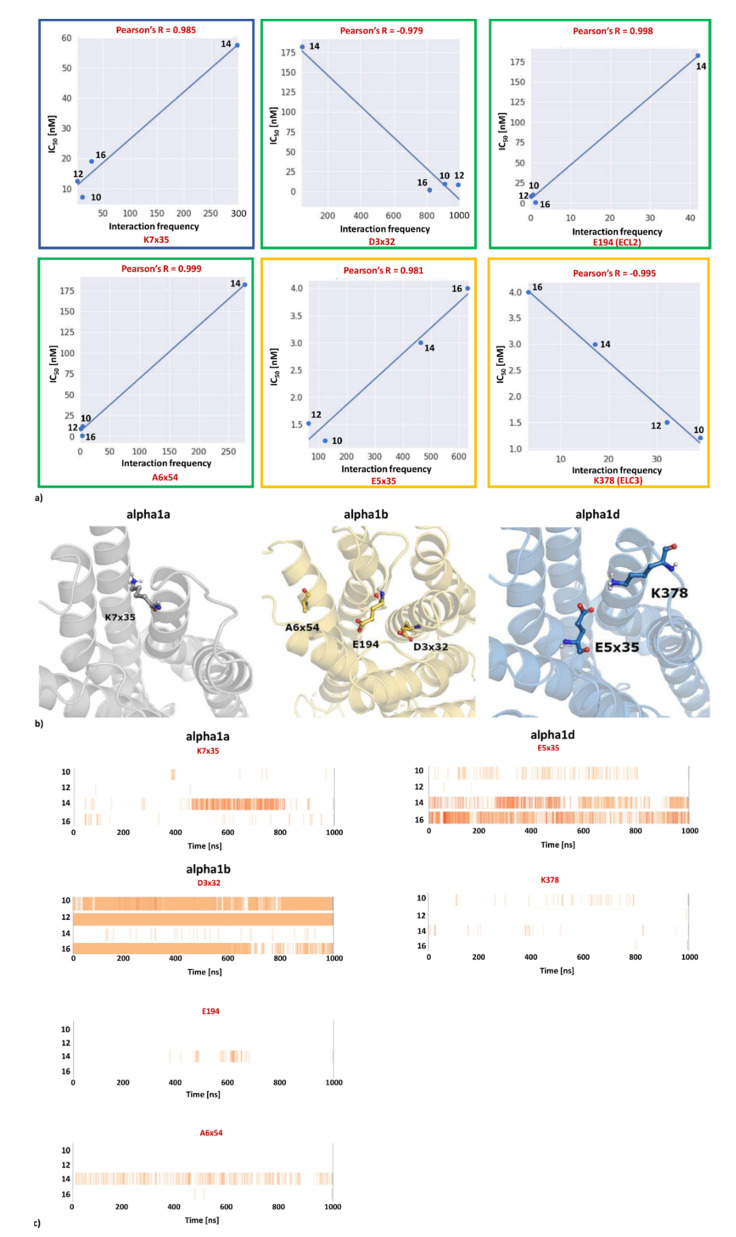
Results of correlation studies (**a**) correlation charts, blue—α_1A_R, green—α_1B_R, yellow—α_1D_R, (**b**) visualization of the most correlated residues, (**c**) contacts of ligands with the most correlated residues which occurred during MD simulation.

In order to examine the interaction patterns obtained in docking in more detail and verify the stability of the obtained docking poses and indicate contacts which might have influence on the observed compound activities, molecular dynamics (MD) simulations were carried out. In addition to the qualitative analysis of the results, the interaction schemes obtained in each MD simulation were quantitatively confronted with experimental data to indicate positions, which can be responsible for the observed activity profiles. The procedure involved the generation of the interaction fingerprints (IFPs) [30] and the calculation of the Pearson correlation [31] coefficient (the correlation was determined between the total number of contacts with a given residue and antagonist potencies gathered in Table 2). The results of this analysis are presented in Figure 6.

The examination of correlations between contact frequency and the outcome of experimental studies indicate that there are positions for which a very high correlation exists. This is reflected not only by high values of the Pearson’s correlation coefficients, but also the examination of the correlation charts and interaction diagrams from MD simulations confirms respective tendencies. 

The highest number of highly correlated residues was indicated for α_1B_R. Interestingly, one representative of these amino acids was the D3 × 32 (contact formed via α_1B_R the piperazine moiety of ligands) position, which is known for the importance of its compound activity. This finding confirms the validity of the approach applied. 

Within the six amino acid positions presented in Figure 6, there are also two that belong to the region of extracellular loops (E194 from ECL2 α_1B_R, and K378 from ECL3 α_1D_R). This indicates the importance of extracellular loops in the specific interaction with ligands and the necessity of the careful consideration of the interaction of compounds with these protein regions when designing new ligands of the desired activity pattern towards α-adrenergic receptors. 

#### 2.3.3. Examination of Activity Profiles towards Serotonin Receptor Subtypes

**Figure 7:**
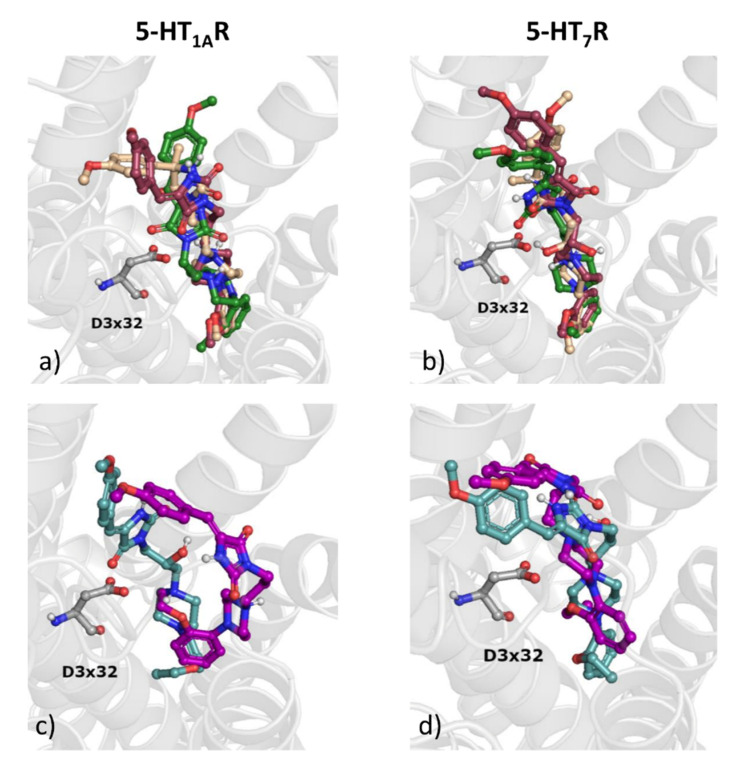
Docking results of selected compounds—**3**—beige, **6**—dark pink, **12**—dark green, **7**—turquoise, **13**—purple to 5-HT_1A_R (**a**,**c**), and 5-HT_7_R (**b**,**d**).

The molecular modeling study of serotonin receptors focused on two main aspects: examination of the influence of the hydroxy group in linker and the influence of the hybridization of C5 of hydantoin moiety on the ligand activity profile. In order to answer these questions, a group of compounds was selected for careful examination: **3**, **6**, **12** and also **7** and **13**. The outcome of the docking studies is presented in Figure 7.

For all analyzed compounds, the ligands are oriented in such a way that the arylpiperazine moiety is deeply buried in the binding pocket. In general, the compounds share similar fitting in the deeper part of the binding cavity, whereas their orientation varies significantly, when considering the upper part of the pocket.

The binding data indicate that the introduction of the hydroxyl group to the linker does not seem to improve compound activity, despite forming strong interactions with respective amino acids. In general, affinity changes when considering OH-substituted ligands and their unsubstituted analogs are higher for 5-HT_1A_R than for 5-HT_7_R. Despite sharing the similar position of the arylpiperazine moiety in 5-HT_1A_R, the hydroxy group makes contacts with different residues. For compound **3**, it forms a hydrogen bond with D3 × 32, for **6**, with Y7 × 42, and for **7**, with N7 × 38. However, although for **12**, the respective contacts formed by the OH group are not present, there are other interactions formed, which strongly keep the compound in the 5-HT_1A_R binding site: a hydrogen bond with N7 × 38 formed by the hydantoin moiety, a hydrogen bond with W7 × 39 formed by the methoxy group and a hydrogen bond with D3 × 32 (contact with the piperazine). In addition, pi-pi stacking is observed with W6 × 48, F5 × 45, and Y2 × 63. A similar situation is observed for **13**, where several hydrogen bonds are also formed despite the lack of a hydroxy group in the linker. It is also worth indicating that, although compound **7** and **13** differ only in terms of the presence/absence of the hydroxy group in the linker, their docking poses are significantly different (higher pose variation again for 5-HT_1A_R, which is also correlated with higher activity change towards this receptor). Interestingly, **7** is located a little bit deeper in the binding pocket of 5-HT_7_R than **13**, whereas the relative position of these two ligands towards D3 × 32 of 5-HT_1A_R is similar. Summarizing, the presence of the hydroxy group in the linker has no significant influence on 5-HT_7_R affinity, whereas it causes a decrease of 5-HT_1A_R affinity, for example, from *K_i_* = 45.8 nM (compound **13**) to *K_i_* = 313 nM (compound **7**). Interestingly, the sp^3^ hybridization of the C5 atom of hydantoin turned out to be a key factor for high 5-HT_7_R affinity, which provides enhanced structural flexibility (*K_i_* = 8 nM for compound **3** and *K_i_* = 197 nM for its analogue stiffened with double bond, i.e., compound **6**). On the other hand, the structural stiffness achieved by the presence of the sp^2^-hybrydized C5 carbon atom provides a significant increase of general α-AR affinity (with *K_i_* = 530 nM for compound **3** and *K_i_* = 144.6 nM for compound **6**).

## 3. Materials and Methods

### 3.1. Chemical Synthesis

^1^H NMR spectra (supplementary materials) for all the final compounds and ^13^C NMR spectra for representative compounds (**7** and **8**) were recorded on a Varian Mercury VX 300 MHz (Varian INC., Palo Alto, CA USA). Chemical shifts are expressed in parts per million (ppm), using the solvent (DMSO) signal as an internal standard. Data are reported using the following abbreviations: s, singlet; bs, broad singlet; d, doublet; t, triplet; q, quartet; p, pentet; Ar, aromatic, Pp, piperazine, Ph, phenyl (As shown in the Supporting Information). Thin-layer chromatography (TLC) was performed on pre-coated Merck silica gel 60 F254 aluminum sheets (Munich, Germany). The mass of compounds was recorded on a Waters ACQUITYTM UPLC (Waters, Milford, MA, USA) coupled to a Waters TQD mass spectrometer (electrospray ionization mode, EDI-tandem quadrupole). Retention times (t_R_) are given in minutes. The UPLC/MS purity of all final compounds was determined (%). 

All four 5-arylidene hydantoin derivatives (**17**–**20**) and alkylated intermediates (**21**–**27**) were already described [25,32,33,34].

#### 3.1.1. (Z)-3-(2-hydroxy-3-(4-(2-methoxyphenyl)piperazin-1-yl)propyl)-5-(3-methoxybenzylidene)imidazolidine-2,4-dione (**4**)

White solid. Yield 63%; mp 163–166 °C; LC/MS^+^ purity: 100%, t_R_ = 4.64, MS calcd for [M + H]^+^: C_25_H_30_N_4_O_5_ *m*/*z*: 466.54, found 467.28. ^1^H NMR (500 MHz, DMSO-d_6_) δ [ppm]: 10.74 (s, 1H, N^1^H), 7.26 (t, *J* = 7.9 Hz, 1H, Ar), 7.16 (d, *J* = 7.6 Hz, 1H, Ar), 7.10 (s, 1H, Ar), 6.85 (s, 2H, Ar), 6.82 (d, *J* = 7.0 Hz, 1H, Ar), 6.79 (d, *J* = 7.1 Hz, 1H, Ar), 6.72–6.68 (m, 1H, Ar), 6.46 (s, 1H, Ph–CH=), 4.96 (br. s, 1H, OH), 3.98–3.96 (m, 1H, CH-OH), 3.73 (s, 3H, OCH_3_), 3.70 (s, 3H, OCH_3_), 3.50–3.48 (m, 2H, N^3^-CH_2_), 2.85 (br. s, 4H, Pp-3,5-H), 2.52 (br. s, 2H, Pp-CH_2_), 2.40–2.34 (m, 4H, Pp-2,6-H).

#### 3.1.2. (Z)-3-(3-(4-(2-ethoxyphenyl)piperazin-1-yl)-2-hydroxypropyl)-5-(3-methoxybenzylidene)imidazolidine-2,4-dione (**5**)

White solid. Yield 41%; mp 120–123 °C; LC/MS^+^ purity: 97.33%, t_R_ = 4.71, MS calcd for [M + H]^+^: C_26_H_32_N_4_O_5_ *m*/*z*: 480.24, found 481.37. ^1^H NMR (500 MHz, DMSO-d_6_) δ [ppm]: 10.84 (s, 1H, N^1^H), 9.96 (br. s, 1H, OH), 7.35–7.26 (m, 1H, Ar), 7.20 (d, *J* = 7.7 Hz, 1H, Ar), 7.13–7.12 (m, 1H, Ar), 6.83–6.94 (m, 5H, Ar), 6.49 (s, 1H, Ph–CH=), 4.29–4.25 (m, 1H, CH-OH), 3.96 (q, *J* = 6.9 Hz, 2H, O-CH_2_-CH_3_), 3.76 (s, 3H, O-CH_3_), 3.64–3.38 (m, 6H, Pp 3,5 H, Pp CH_2_), 3.36–2.91 (m, 6H, Pp 2,6 H, N^3^-CH_2_), 1.30 (t, *J* = 7.0 Hz, 3H, O-CH_2_-CH_3_).

#### 3.1.3. (Z)-3-(2-hydroxy-3-(4-(2-methoxyphenyl)piperazin-1-yl)propyl)-5-(4-methoxybenzylidene)imidazolidine-2,4-dione (**6**)

White solid. Yield 57%; mp 176–178 °C; LC/MS^+^ purity: 100%, t_R_ = 4.27, MS calcd for [M + H]^+^: C_25_H_30_N_4_O_5_ *m*/*z*: 466.22, found 467.21. ^1^H NMR (500 MHz, DMSO-d_6_) δ [ppm]: 10.61 (s, 1H, N^1^H), 7.55 (d, *J* = 8.8 Hz, 2H, Ar), 6.91 (d, *J* = 8.9 Hz, 2H, Ar), 6.88–6.82 (m, 2H, Ar), 6.77– 6.64 (m, 2H, Ar), 6.46 (s, 1H, Ph–CH=), 4.95 (br. s, 1H, OH), 4.02–3.90 (m, 1H, CH-OH), 3.74 (s, 3H, OCH_3_), 3.70 (s, 3H, OCH_3_), 3.55–3.35 (m, 2H, N^3^-CH_2_), 2.97–2.71 (m, 4H, Pp-3,5-H), 2.63–2.47 (m, 2H, Pp-CH_2_), 2.46–2.32 (m, 4H, Pp-2,6-H).

#### 3.1.4. (Z)-3-(3-(4-(2-ethoxyphenyl)piperazin-1-yl)-2-hydroxypropyl)-5-(4-methoxybenzylidene)imidazolidine-2,4-dione (**7**)

White solid. Yield 53%; mp 180–183 °C; LC/MS^+^ purity: 100%, t_R_ = 4.64, MS calcd for [M + H]^+^: C_26_H_32_N_4_O_5_ *m*/*z*: 480.24, found 481.30. ^1^H NMR (500 MHz, DMSO-d_6_) δ 10.65 (br s, 1H, N^1^H), 7.57 (d, *J* = 8.75 Hz, 2H, Ar), 6.90 (d, *J* = 8.8 Hz, 2H, Ar), 6.83–6.80 (m, 2H, Ar), 6.75–6.70 (m, 1H, Ar), 6.69–6.67 (m, 1H, Ar), 6.46 (s, 1H, Ph–CH=), 4.94 (s, 1H, OH), 3.99–3.97 (m, 1H, CH-OH), 3.91 (q, *J* = 6.9 Hz, 2H, OCH_2_-CH_3_), 3.75 (s, 3H, OCH_3_), 3.58–3.39 (m, 2H, N^3^-CH_2_), 2.93–2.82 (m, 4H, Pp-3,5-H), 2.52–2.58 (m, 2H, Pp-CH_2_), 2.40–2.26 (m, 4H, Pp-2,6-H), 1.27 (t, *J* = 6.9 Hz, 3H, O-CH_2_-CH_3_). ^13^C NMR (125 MHz, DMSO-d_6_) δ [ppm]: 165.04, 160.02, 155.93, 151.66, 141.96, 131.66, 125.96, 125.52, 122.69, 121.35, 118.37, 114.84, 113.67, 109.91, 64.09, 63.76, 63.53, 55.80, 54.19, 50.39, 44.10, 15.35.

#### 3.1.5. (Z)-3-(3-(4-(2-fluorophenyl)piperazin-1-yl)-2-hydroxypropyl)-5-(4-methoxybenzylidene)imidazolidine-2,4-dione (**8**)

White solid. Yield 62%; mp 104–106 °C; LC/MS^+^ purity: 100%, t_R_ = 4.36, MS calcd for [M + H]^+^: C_24_H_27_FN_4_O_4_ *m*/*z*: 454.20, found 455.18. ^1^H NMR (500 MHz, DMSO-d_6_) δ [ppm]: 10.59 (s, 1H, N^1^H), 7.55 (d, *J* = 8.6 Hz, 2H, Ar), 7.06–7.02 (m, 1H, Ar), 6.98–6.95 ( t, *J* = 7.6 Hz, 1H Ar), 6.91 (d, *J* = 8.6 Hz, 2H, Ar), 6.89–6.86(m, 1H, Ar), 6.83 (t, *J* = 8.4 Hz, 1H, Ar) 6.46 (s, 1H, Ph–CH=), 4.95–4.94 (m, 1H, OH), 3.97–3.96 (m, 1H, CH-OH), 3.75 (s, 3H, OCH_3_), 3.61–3.40 (m, 2H, N^3^-CH_2_), 2.90–2.88 (m, 4H, Pp-3,5-H), 2.60–2.50 (m, 2H, Pp-CH_2_), 2.44–2.27 (m, 4H, Pp-2,6-H).^13^C NMR (125 MHz, DMSO-d_6_ ) δ [ppm]: 165.04, 160.02, 156.46, 155.94, 154.52, 140.44, 140.37, 131.66, 125.95, 125.51, 125.23, 125.20, 119.66, 119.64, 116.44, 116.28, 114.83, 109.94, 64.11, 63.40, 53.95, 55.58, 50.56, 44.08.

#### 3.1.6. (Z)-3-(3-(4-(2-ethoxyphenyl)piperazin-1-yl)-2-hydroxypropyl)-5-(3,4,5-trimethoxybenzylidene)imidazolidine-2,4-dione (**9**)

White solid. Yield 62%; mp 104–106 °C; LC/MS^+^ purity: 100%, t_R_ = 4.59, MS calcd for [M + H]^+^: C_28_H_36_N_4_O_7_ *m*/*z*: 540.62, found 541.25. ^1^H NMR (500 MHz, DMSO-d_6_) δ [ppm]: 10.78 (s, 1H, N^1^H), 6.83–6.82 (m, 2H, Ar), 6.81 (s, 2H, Ar), 6.78–6.67 (m, 2H, Ar), 6.45 (s, 1H, Ph–CH=), 4.96–4.95 (d, *J* = 5.4 Hz, 1H, OH), 3.99–3.97 (m, 1H, CH-OH), 3.92 (q, *J* = 7.0 Hz, 2H, O-CH_2_-CH_3_), 3.76 (s, 6H, 2xOCH_3_), 3.63 (s, 3H, OCH_3_), 3.51–3.47 (m, 2H, N^3^-CH_2_), 3.28–3.24 (m, 2H, Pp-CH_2_), 2.97–2.76 (m, 4H, Pp-3,5-H), 2.43–2.27 (m, 4H, Pp-2,6-H), 1.26 (t, *J* = 7.0 Hz, 3H, O-CH_2_-CH_3_).

#### 3.1.7. (Z)-3-(3-(4-(2-ethoxyphenyl)piperazin-1-yl)propyl)-5-(3-methoxybenzylidene)imidazolidine-2,4-dione (**10**)

White solid. Yield 24%; mp 120–122 °C; LC/MS^+^ purity: 100%, t_R_ = 4.70, MS calcd for [M + H]^+^: C_26_H_32_N_4_O_4_ *m*/*z*: 464.24, found 465.37. ^1^H NMR (500 MHz, DMSO-d_6_) δ [ppm]: 10.74 (s, 1H, N^1^H), 7.28–7.24 (m, 1H, Ar), 7.16 (d, *J* = 6.9 Hz, 1H, Ar), 7.10–7.09 (m, 1H, Ar), 6.87–6.85 (m, 1H, Ar), 6.83–6.82 (m, 2H, Ar), 6.71–6.81 (m, 2H, Ar), 6.45 (s, 1H, Ph–CH=), 3.94 (q, *J* = 7.0 Hz, 2H, O-CH_2_-CH_3_), 3.74 (s, 3H, O-CH_3_), 3.52–3.45 (m, 2H, N^3^-CH_2_), 2.97–2.72 (m, 4H, Pp-3,5-H), 2.62–2.49 (m, 2H, CH_2_), 2.42–2.27 (m, 6H, Pp-2,6-H, Pp-CH_2_), 1.28 (t, *J* = 6.9 Hz, 3H, O-CH_2_-CH_3_).

#### 3.1.8. (Z)-3-(3-(4-(2-chlorophenyl)piperazin-1-yl)propyl)-5-(3-methoxybenzylidene)imidazolidine-2,4-dione (**11**)

White solid. Yield 31%; mp 144–146 °C; LC/MS^+^ purity: 96.78%, t_R_ = 4.93, MS calcd for [M + H]^+^: C_24_H_27_ClN_4_O_3_ *m*/*z*: 454.18, found 455.25. ^1^H NMR (500 MHz, DMSO-d_6_) δ [ppm]: 10.77 (s, 1H, N^1^H), 7.32 (dd, *J* = 7.9, 1.5 Hz, 1H, Ar), 7.26 (t, *J* = 7.9 Hz, 1H, Ar), 7.18 (d, *J* = 7.7 Hz, 1H, Ar), 7.14 (d, *J* = 3.3 Hz, 1H, Ar), 7.11 (dd, *J* = 7.8, 1.7 Hz, 1H, Ar), 6.96–6.92 (m, 2H, Ar), 6.86 (dd, *J* = 8.6, 2.9 Hz, 1H, Ar), 6.44 (s, 1H, Ph–CH=), 3.74 (s, 3H, O-CH_3_), 3.53 (t, *J* = 6.7 Hz, 2H, N^3^-CH_2_), 2.88–2.86 (m, 4H, Pp-3,5-H), 2.45–2.39 (m, 4H, Pp-2,6-H), 2.35 (t, *J* = 6.5 Hz, 2H, Pp-CH_3_), 1.72 (p, *J* = 6.5 Hz, 2H, CH_2_).

#### 3.1.9. (Z)-5-(4-methoxybenzylidene)-3-(3-(4-(2-methoxyphenyl)piperazin-1-yl)propyl)imidazolidine-2,4-dione (**12**)

White solid. Yield 57%; mp 176–179 °C; LC/MS^+^ purity: 96.78%, t_R_ = 4.52, MS calcd for [M + H]^+^: C_25_H_30_N_4_O_4_ *m*/*z*: 450.23, found 451.26. ^1^H NMR (500 MHz, DMSO-d_6_) δ [ppm]: 7.67 (d, *J* = 8.4 Hz, 2H, Ar), 6.93–6.82 (m, 5H, Ar), 6.81–6.71 (m, 2H, Ar, Ph-CH=), 3.73 (s, 3H, O-CH_3_), 3.71 (s, 3H, O-CH_3_), 3.42–3.40 (m, 2H, N^3^-CH_2_), 2.86–2.83 (m, 4H, Pp-3,5-H), 2.41–2.39 (m, 4H, Pp-2,6-H), 2.33–2.28 (m, 2H, Pp-CH_2_), 1.73–1.60 (m, 2H, CH_2_). 

#### 3.1.10. (Z)-3-(3-(4-(2-ethoxyphenyl)piperazin-1-yl)propyl)-5-(4-methoxybenzylidene)imidazolidine-2,4-dione (**13**)

White solid. Yield 53%; mp 180–183 °C; LC/MS^+^ purity: 100%, t_R_ = 4.90, MS calcd for [M + H]^+^: C_26_H_32_N_4_O_4_ *m*/*z*: 464.24, found 465.35. ^1^H NMR (500 MHz, DMSO-d_6_) δ [ppm]: 10.60 (s, 1H, N^1^H), 7.56 (d, *J* = 9.7 Hz, 2H, Ar), 6.91 (d, *J* = 8.9 Hz, 2H, Ar), 6.85–6.83 (m, 2H, Ar), 6.75–6.68 (m, 2H, Ar), 6.47 (s, 1H, Ph–CH=), 3.94 (q, *J* = 7.0 Hz, 2H, O-CH_2_-CH_3_), 3.75 (s, 3H, O-CH_3_), 3.51 (t, *J* = 6.9 Hz, 2H, N^3^-CH_2_), 3.26–3.24 (m, 4H, Pp-3,5-H), 2.94–2.80 (m, 4H, Pp-2,6-H), 2.32 (t, *J* = 7.0 Hz, 2H, Pp-CH_2_), 1.71 (p, *J* = 7.0 Hz, 2H, CH_2_), 1.27 (t, *J* = 7.0 Hz, 3H, O-CH_2_-CH_3_).

#### 3.1.11. (Z)-3-(3-(4-(2-methoxyphenyl)piperazin-1-yl)propyl)-5-(3,4,5-trimethoxybenzylidene)imidazolidine-2,4-dione (**15**)

White solid. Yield 20%; mp 130–132 °C; LC/MS^+^ purity: 100%, t_R_ = 4.48, MS calcd for [M + H]^+^: C_27_H_34_N_4_O_6_ *m*/*z*: 510.25, found 511.21. ^1^H NMR (500 MHz, DMSO-d_6_) δ [ppm]: 10.77 (s, 1H, N^1^H), 6.87–6.84 (m, 2H, Ar), 6.82 (s, 2H, Ar), 6.73–6.71 (m, 2H, Ar), 6.46 (s, 1H, Ph–CH=), 3.76 (s, 6H, 2xO-CH_3_), 3.70 (s, 3H, O-CH_3_), 3.63 (s, 3H, O-CH_3_), 3.53 (t, *J* = 6.7 Hz, 2H, N^3^–CH_2_), 2.86–2-84 (m, 4H, Pp-3,5-H), 2.43–2.32 (m, 6H, Pp-2,6-H, Pp-CH_2_), 1.72 (p, *J* = 6.6 Hz, 2H, CH_2_).

#### 3.1.12. (Z)-3-(3-(4-(2-ethoxyphenyl)piperazin-1-yl)propyl)-5-(3,4,5-trimethoxybenzylidene)imidazolidine-2,4-dione (**16**)

White solid. Yield 20%; mp 130–132 °C; LC/MS^+^ purity: 100%, t_R_ = 4.87, MS calcd for [M + H]^+^: C_28_H_36_N_4_O_6_ *m*/*z*: 524.26, found 525.30. ^1^H NMR (500 MHz, DMSO-d_6_) δ [ppm]: 10.80 (s, 1H, N^1^H), 6.97–6.83 (m, 6H, Ar), 6.49 (s, 1H, Ph–CH=), 3.99 (q, *J* = 7.0 Hz, 2H, O-CH_2_-CH_3_), 3.81 (s, 6H, 2xO-CH_3_), 3.65 (s, 3H, O-CH_3_), 3.55–3.49 (m, 6H, N^3^-CH_2_, Pp-3,5-H), 3.20–3.13 (m, 4H, Pp-2,6-H), 2.92–2.87 (m, 2H, Pp-CH_2_), 2.01–1.98 (m, 2H, CH_2_), 1.31 (t, *J* = 6.9 Hz, 3H, O-CH_2_-CH_3_).

### 3.2. Pharmacology In Vitro

#### 3.2.1. Radioligand Binding Assays: Affinity for α_1_-Receptor

Tissue (rat cortex) was homogenized in 20 volumes of ice-cold 50 mM Tris-HCl buffer, pH 7.6, using an Ultra Turrax T25B homogenizer (IKA, Staufen, Germany). The homogenate was centrifuged at 20,000× *g* for 20 min. The resulting supernatant was decanted and pellet was resuspended in the same buffer and centrifuged again in the same conditions. The final pellet was resuspended in an appropriate volume of buffer (10 mg/1 mL). 

[^3^H]Prazosin (spec. act. 85 Ci/mmol, Perkin Elmer) was used for labeling the α_1_-receptor and 240 μL of the tissue suspension, 30 μL of 10 μM phentolamine (displacer), 30 μL of 0.2 nM [^3^H]Prazosin and 30 μL of the analyzed compound were incubated at 30 °C for 30 min. The concentrations of analyzed compounds ranged from 10^−10^ to 10^−5^ M. 

The incubation was terminated by rapid filtration over glass fiber filters FilterMate B (PerkinElmer, Waltham, MA, USA) using a 96-well FilterMate harvester (PerkinElmer, Waltham, MA, USA). Five rapid washes were performed with ice-cold 50 mM Tris-HCl buffer, pH 7.6.

The FilterMate was dried in a microwave and placed in a plastic bag (PerkinElmer, Waltham, MA, USA) and soaked in 10 mL of a liquid scintillation cocktail Ultima Gold MV (PerkinElmer, Waltham, MA, USA). After even distribution of the scintillation cocktail, the filter bag was sealed.

The radioactivity on the filter was measured by a MicroBeta TriLux 1450 scintillation counter (PerkinElmer, Waltham, MA, USA).

Radioligand binding data were analyzed using iterative curve fitting routines GraphPad Prism 3.0 (GraphPad Software, San Diego, CA, USA) using the built-in three parameter logistic model describing ligand competition binding to radioligand-labeled sites. The log IC_50_ (i.e., the log of the ligand concentration that reduces specific radioligand binding by 50%) estimated from the data is used to obtain the *K*_i_ by applying the Cheng–Prusoff approximation [35].

#### 3.2.2. Radioligand Binding Assays: Binding Affinities for 5-HT_1A_, 5-HT_6_ and 5-HT_7_ Receptors

HEK-293 cells with a stable expression of human 5-HT_1A_, 5-HT_6_ and 5-HT_7b_ receptors (prepared with the use of Lipofectamine 2000) were maintained at 37 °C in a humidified atmosphere with 5% CO_2_ and grown in Dulbecco’s Modifier Eagle Medium containing 10% dialyzed fetal bovine serum and 500 μg/mL G418 sulfate. For membrane preparation, cells were subcultured in 150 cm^3^ flasks, grown to 90% confluence, washed twice with phosphate buffered saline (PBS) prewarmed to 37 °C and pelleted by centrifugation (200× *g*) in PBS containing 0.1 mM EDTA and 1 mM dithiothreitol. Prior to membrane preparation, pellets were stored at −80 °C. Cell pellets were thawed and homogenized in 10 volumes of assay buffer using an Ultra Turrax tissue homogenizer and were centrifuged twice at 35,000× *g* for 15 min at 4 °C, with incubation for 15 min at 37 °C in-between. The composition of the assay buffers was as follows: for 5-HT_1A_R: 50 mM Tris HCl, 0.1 mM EDTA, 4 mM MgCl_2_, 10 μM pargyline and 0.1% ascorbate, for 5-HT_6_R—50mM TrisHCl, 0.5 mM EDTA and 4 mM MgCl_2_; and for 5- HT_7b_R: 50 mM Tris HCl, 4 mM MgCl_2_, 10 μM pargyline and 0.1% ascorbate. All the assays were incubated in a total volume of 200 μL in 96-well microtiter plates for 1 h at 37 °C, except those for 5-HT_1A_R, which were incubated at room temperature. The process of equilibration was terminated by rapid filtration through Unifilter plates with a FilterMate Unifilter 96 Harvester (PerkinElmer, USA). The radioactivity bound to the filters was quantified on a Microbeta TopCount instrument (PerkinElmer, USA). For competitive inhibition studies, the assay samples contained the following as radioligands (PerkinElmer, USA): 2.5 nM [^3^H]-8-OH-DPAT (135.2 Ci/mmol) for 5-HT_1A_R; 2 nM [^3^H]-LSD (83.6 Ci/mmol) for 5-HT_6_R, and 0.8 nM [^3^H]-5-CT (39.2 Ci/mmol) for 5-HT_7_R. Non–specific binding was defined with 10 μM of 5-HT in 5-HT_1A_R and 5-HT_7_R binding experiments, whereas 10 mM of methiothepin was used in the 5-HT_6_R assays. Each compound was tested in triplicate at seven concentrations (10^–10^–10^−4^M). The inhibition constants (*K_i_*) were calculated from the Cheng–Prusoff equation [35]. For all the binding assays, results were expressed as the means of at least two separate experiments.

### 3.3. Functional Tests

#### 3.3.1. Determination of the Intrinsic Activity for the α_1A_-Ars

An intrinsic activity assay was performed according to the manufacturer of the assay kit (Invitrogen, Thermo Fisher Scientific). The cells were harvested and suspended in Assay Medium to a density of 312,500 cells/mL. Of the cell suspension, 32 µL per well was added to the Test Compound wells, the Unstimulated Control wells, and Stimulated Control wells and were incubated per 16–24 h. To perform an agonist assay, eight concentrations of 8 µL of the tested compound (10^−4^–10^−11^ M), for example, in five-fold higher concentration in comparison to the final tested concentration in the well, were added to the cells. To perform an antagonist assay, eight concentrations of 4 µL of the tested compound (10^−4^–10^−11^ M), for example, in a ten-fold higher concentration in comparison to the final tested concentration in the well, were added to the cells. Then, after 30 min, 4 µL of a standard agonist, phenylephrine, in EC_80_ (ten-fold higher concentration in comparison to the EC_80_ in the well), in Assay Medium, was added to the cells. Then, both the agonist and the antagonist plate were incubated in a humidified 37 °C/5% CO_2_ incubator for 5 h. Then, cells were loaded with 8 µL of LiveBLAzer™-FRET B/G Substrate Mixture (CCF4-AM, Thermo Fisher Scientific) and incubated at room temperature for 2 h.

#### 3.3.2. Determination of the Intrinsic Activity for the α_1B_-ARs and α_1D_-Ars

The Aequoscreen technology uses the recombinant cell lines with stable expression of the α_1B_ or α_1D_ adrenoreceptor and co-expression of apoaequorin and a GPCR as a system to detect the activation of the receptor, following the addition of an agonist, via the measurement of light emission. For measurement, cells (frozen, ready to use) were thawed and resuspended in 10-mL of assay buffer containing 5 μM coelenterazine h. This cell suspension was put in a 10-mL Falcon tube, fixed onto a rotating wheel and incubated overnight at RT° in the dark. Cells were diluted with Assay Buffer to 5000 cells/20 µL. In the first part, agonistic activity was tested. Potential agonists (standard and tested) 2× (50 μL/well), diluted in Assay Buffer, were prepared in 1/2 white polystryrene area plates, and the cell suspension was dispensed in 50 μL volume on the ligands using the injector. The light emitted was recorded for 20 s. In the second part, the agonistic activity was tested. Cells with tested compounds were incubated for 15 min at room temperature. Therefore, 50 µL of standard agonist, phenylephrine (3 × EC80 final concentration) was injected into the mix of cells and the antagonist, and the light emitted was recorded for 20 s.

### 3.4. Molecular Modeling and Cheminformatic Analysis

#### 3.4.1. Searches for Structurally Similar Compounds in the ChEMBL Database

The ChEMBL database v.26 was used as a source of all compounds with experimentally confirmed activity towards the considered targets. All data records referring to 5-HT_1A_R, 5-HT_6_R, 5-HT_7_R, α_1A_R, α_1B_R, and α_1D_R (human-related data) were downloaded. Targets with the following ChEMBLIDs were considered: CHEMBL214 (5-HT_1A_R), CHEMBL3371 (5-HT_6_R), CHEMBL3155 (5-HT_7_R), CHEMBL229 (α_1A_R), CHEMBL232 (α_1B_R) and CHEMBL223 (α_1D_R). The similarity coefficients for all examined compounds towards all the above-described ChEMBL data were determined (Tanimoto similarity was used [28], and ECFP [36] was used for compounds representation) using the InstantJChem v. 16.12.5.0 software [37].

#### 3.4.2. Docking

The docking was performed with the use of the GPCRdb homology models of the respective receptors (inactive receptor states were considered). Receptors were prepared for docking using the Protein Preparation Wizard from the Schrodinger Suite. The compounds were prepared for docking using LigPrep [38] (protonation states for pH 7.4 were generated) and were docked to the considered receptor models in Glide [39] (grid centering on the aspartic acid from the third transmembrane helix; D3 × 32 according to the GPCRdb numbering) at extra precision mode. The obtained ligand-receptor complexes with the best docking score constituted an input for MD simulations.

#### 3.4.3. Molecular Dynamics

MD simulations were carried out in Desmond [40] using the TIP3P solvent model [41] and POPC (palmitoyl-oleil-phosphatidylcoline) as a membrane model (force-field: OPLS3e, pressure: 1.01325 bars, temperature: 300 K). The box shape was orthorhombic of the size of 10 Å × 10 Å × 10 Å. In each case, the system was neutralized by the addition of the respective number of Cl- ions and relaxed before the simulation; the duration of each simulation was equal to 1000 ns.

## 4. Conclusions

The work presented here concerns the deep structural insight into the hydantoin-arylpiperazine group and their serotonin/α-adrenergic activity. The computer-aided SAR analysis enabled us to answer the questions about the influence of particular structural fragments on selective vs. multifunctional activity. The obtained results led to conclusions that the hybridization type of the C5 carbon atom of hydantoin and the presence of a hydroxy group in the linker are key structural determinants for balancing between serotonin and α-adrenergic affinity. The selectivity among adrenergic subtypes turned out to be the most challenging issue; however, the results clearly indicate the importance of extracellular loops, which should be considered during the design of novel molecules. 

As a result of the performed investigations, the two lead structures that were selected for the further studies were compounds **12** and **14**. Compound **12** is a very promising ‘lead’ for the search of novel multifunctional anxiolytic agents, as it possesses high multifunctional α_1A_/α_1D_-AR/5-HT_1A_R activity and moderates 5-HT_7_R affinity. Compound **14**, despite its lower serotoninergic affinity, has high α_1A_/α_1D_ affinity and moderates selectivity towards α_1B_, which is recommended due to the elimination of probable cardiotoxic effects [4]. The structural conclusions of this work provide meaningful support for future lead optimization in order to achieve the desired pharmacodynamic profile in the search for new CNS-modulating agents. 

## Figures and Tables

**Table 1:**
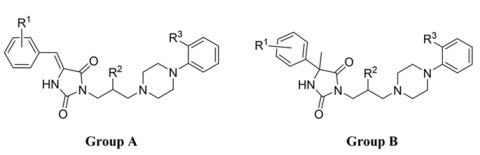
Radioligand binding assays results for compounds **1**–**16**.

Cmpd	Group	R ^1^	R ^2^	R ^3^	*K_i_* [nM]			
					α_1_	5-HT_1A_	5-HT_6_	5-HT_7_
1 *	A	3,4-diOCH_3_	OH	2-OCH_3_	44.5	nt	nt	nt
2 *	B	4-F	OH	2-OCH_3_	230	121	10,790	3
3 *	B	4-OCH_3_	OH	2-OCH_3_	530	99	nt	8
**4**	A	3-OCH_3_	OH	2-OCH_3_	187.0 ± 18.6	530	17,610	283
**5**	A	3-OCH_3_	OH	2-OC_2_H_5_	75.9 ± 2.3	556.7	19,220	79
**6**	A	4-OCH_3_	OH	2-OCH_3_	144.6 ± 13.3	282	20,420	197
**7**	A	4-OCH_3_	OH	2-OC_2_H_5_	99.1 ± 11.3	313	10,250	120
**8**	A	4-OCH_3_	OH	2-F	143.7 ± 7.9	3532	11,730	1036
**9**	A	3,4,5-triOCH_3_	OH	2-OC_2_H_5_	89.5 ± 9.0	1870	2835	1253
**10**	A	3-OCH_3_	H	2-OC_2_H_5_	20.2 ± 4.1	103.0	nt	nt
**11**	A	3-OCH_3_	H	2-Cl	44.8 ± 3.3	319	1720	152
**12**	A	4-OCH_3_	H	2-OCH_3_	34.6 ± 4.6	20.6	21,990	101
**13**	A	4-OCH_3_	H	2-OC_2_H_5_	19.5 ± 2.6	45.8	>50,000	114
**14**	A	3,4-diOCH_3_	H	2-OCH_3_	11.9 ± 2.2	128	15,780	189
**15**	A	3,4,5-triOCH_3_	H	2-OCH_3_	25.8 ± 2.9	172	12,320	668
**16**	A	3,4,5-triOCH_3_	H	2-OC_2_H_5_	14.7 ± 1.1	164.5	15,240	138
**Ref**	-	-	-	-	15.7 ± 1.5 ^1^	32 ^2^	7 ^3^	62 ^4^

^*^ Previously reported arylpiperazine hydantoin derivatives [25,26]; ^1^ fentolamine, ^2^ buspirone, ^3^ olanzapine, ^4^ clozapine.

**Table 2:** Functional activity results of reference and tested compounds **10**, **12**, **14** and **16** at α_1A_-AR in rat tail artery, at α_1B_-AR in mouse spleen and at α_1D_-AR in rat aorta.

Cmpd	IC_50_ ± SEM [nM] *		
	α_1A_-AR	α_1B_-AR	α_1D_-AR
**Phenylephrine**	30.0 ± 3.4	27.0 ± 4.2	26.2 ± 4.1
**Prazosin**	4.1 ± 0.5	3.82 ± 0.5	n.d
**Terazosin**	51.9 ± 0.8	1.73 ± 0.3	0.1 ± 0.02
**Tamsulosin**	0.07 ± 0.01	0.13 ± 0.02	n.d.
**10**	7.2 ± 1.1	9.2 ± 1.3	1.2 ± 0.2
**12**	12.8 ± 2.5	8.5 ± 1.1	1.5 ± 0.3
**14**	57.6 ± 7.2	182 ± 21.4	3.0 ± 0.5
**16**	19.1 ± 3.4	0.82 ± 0.1	4.0 ± 0.5

* Antagonist potency was presented as IC_50_ ± SEM, IC_50_ values which were obtained from the linear regression of Schild plot, each value ± SEM of 4–7 experimental results.

## Data Availability

Not applicable.

## Supplementary Materials

The following are available online, Figures S1–S11: 1H NMR and ^13^C NMR spectra of newly synthesized final compounds.

## Sample Availability

Samples of the compounds are not available from the authors.

## References

[1] O’Connell T.D., Jensen B.C., Baker A.J., Simpson P.C. (2014). Cardiac alpha1-adrenergic receptors: Novel aspects of expression, signaling mechanisms, physiologic function, and clinical importance. Pharmacol. Rev..

[2] Morrow A.L., Creese I. (1986). Characterization of alpha 1-adrenergic receptor subtypes in rat brain: A reevaluation of [3H]WB4104 and [3H]prazosin binding. Mol. Pharmacol..

[3] Schwinn D.A., Roehrborn C.G. (2008). α1-Adrenoceptor subtypes and lower urinary tract symptoms. Int. J. Urol..

[4] De Mey C., Michel M.C., McEwen J., Moreland T. (1998). A double-blind comparison of terazosin and tamsulosin on their differential effects on ambulatory blood pressure and nocturnal orthostatic stress testing. Eur. Urol..

[5] Newton T.F., De La Garza R., Brown G., Kosten T.R., Mahoney J.J., Haile C.N. (2012). Noradrenergic α1 Receptor Antagonist Treatment Attenuates Positive Subjective Effects of Cocaine in Humans: A Randomized Trial. PLoS ONE..

[6] Shorter D.I., Zhang X., Domingo C.B., Nielsen E.M., Kosten T.R., Nielsen D.A. (2020). Doxazosin treatment in cocaine use disorder: Pharmacogenetic response based on an alpha-1 adrenoreceptor subtype D genetic variant. Am. J. Drug Alcohol Abuse.

[7] Chen H., Qian Y., Jia H., Yu Y., Zhang H., Zhao S., Shen J. (2020). Synthesis and pharmacological evaluation of naftopidil-based arylpiperazine derivatives containing the bromophenol moiety. Pharmacol. Rep..

[8] Perez D.M., Doze V.A. (2011). Cardiac and neuroprotection regulated by α1-adrenergic receptor subtypes. J. Recept. Signal Transduct..

[9] Breen A., Blankley K., Fine J. (2017). The efficacy of prazosin for the treatment of posttraumatic stress disorder nightmares in U.S. military veterans. J. Am. Assoc. Nurse Pract..

[10] Zhang X., Norton J., Carrière I., Ritchie K., Chaudieu I., Ryan J., Ancelin M.L. (2017). Preliminary evidence for a role of the adrenergic nervous system in generalized anxiety disorder. Sci. Rep..

[11] Takamura N., Masuda T., Inoue T., Nakagawa S., Koyama T. (2012). Progress in Neuro-Psychopharmacology & Biological Psychiatry The effects of the co-administration of the α 1-adrenoreceptor antagonist prazosin on the anxiolytic effect of citalopram in conditioned fear stress in the rat. Prog. Neuropsychopharmacol. Biol. Psychiatry.

[12] Azevedo H., Ferreira M., Costa R.W., Russo V., Russo E., Mascarello A., Guimarães C.R.W. (2019). Preclinical characterization of ACH-000029, a novel anxiolytic compound acting on serotonergic and alpha-adrenergic receptors. Prog. Neuropsychopharmacol. Biol. Psychiatry.

[13] Celada P., Bortolozzi A., Artigas F. (2013). Serotonin 5-HT1A receptors as targets for agents to treat psychiatric disorders: Rationale and current status of research. CNS Drugs.

[14] Overstreet D.H., Commissaris R.C., De la Garza R., File S.E., Knapp D.J., Seiden L.S. (2003). Involvement of 5-HT1A receptors in animal tests of anxiety and depression: Evidence from genetic models. Stress.

[15] Weisstaub N.V., Zhou M., Lira A., Lambe E., González-Maeso J., Hornung J.P., Sibille E., Underwood M., Itohara S., Dauer W.T. (2006). Cortical 5-HT2A receptor signaling modulates anxiety-like behaviors in mice. Science.

[16] Wesołowska A., Nikiforuk A., Stachowicz K., Tatarczyńska E. (2006). Effect of the selective 5-HT7 receptor antagonist SB 269970 in animal models of anxiety and depression. Neuropharmacology.

[17] Nikiforuk A. (2015). Targeting the Serotonin 5-HT 7 Receptor in the Search for Treatments for CNS Disorders: Rationale and Progress to Date. CNS Drugs.

[18] Wróbel M.Z., Chodkowski A., Marciniak M., Dawidowski M., Maksymiuk A., Siwek A., Nowak G., Turło J. (2020). Synthesis of new 4-butyl-arylpiperazine-3-(1H-indol-3-yl)pyrrolidine-2,5-dione derivatives and evaluation for their 5-HT1A and D2 receptor affinity and serotonin transporter inhibition. Bioorg. Chem..

[19] Shimoda Y., Yui J., Xie L., Fujinaga M., Yamasaki T., Ogawa M., Nengaki N., Kumata K., Hatori A., Kawamura K. (2013). Synthesis and evaluation of 1-[2-(4-[11C]methoxyphenyl)phenyl] piperazine for imaging of the serotonin 5-HT7 receptor in the rat brain. Bioorganic Med. Chem..

[20] Szczepańska K., Karcz T., Siwek A., Kuder K.J., Latacz G., Bednarski M., Szafarz M., Hagenow S., Lubelska A., Olejarz-Maciej A. (2019). Structural modifications and in vitro pharmacological evaluation of 4-pyridyl-piperazine derivatives as an active and selective histamine H3 receptor ligands. Bioorg. Chem..

[21] Handzlik J., Bojarski A.J., Satała G., Kubacka M., Sadek B., Ashoor A., Siwek A., Więcek M., Kucwaj K., Filipek B. (2014). SAR-studies on the importance of aromatic ring topologies in search for selective 5-HT 7 receptor ligands among phenylpiperazine hydantoin derivatives. Eur. J. Med. Chem..

[22] Löber S., Aboul-Fadl T., Hübner H., Gmeiner P. (2012). Di- and trisubstituted pyrazolo[1,5-a]pyridine derivatives: Synthesis, dopamine receptor binding and ligand efficacy. Bioorganic Med. Chem. Lett..

[23] Hayatshahi H.S., Xu K., Griffin S.A., Taylor M., Mach R.H., Liu J., Luedtke R.R. (2018). Analogues of Arylamide Phenylpiperazine Ligands to Investigate the Factors Influencing D3 Dopamine Receptor Bitropic Binding and Receptor Subtype Selectivity. ACS Chem. Neurosci..

[24] Handzlik J., Szymańska E., Wójcik R., Dela A., Jastrzebska-Wiesek M., Karolak-Wojciechowska J., Fruziński A., Siwek A., Filipek B., Kieć-Kononowicz K. (2012). Synthesis and SAR-study for novel arylpiperazine derivatives of 5-arylidenehydantoin with α 1-adrenoceptor antagonistic properties. Bioorganic Med. Chem..

[25] Czopek A., Bucki A., Kołaczkowski M., Zagórska A., Drop M., Pawłowski M., Siwek A., Głuch-Lutwin M., Pękala E., Chrzanowska A. (2019). Novel multitarget 5-arylidenehydantoins with arylpiperazinealkyl fragment: Pharmacological evaluation and investigation of cytotoxicity and metabolic stability. Bioorganic Med. Chem..

[26] Kucwaj-Brysz K., Kurczab R., Jastrzębska-Więsek M., Żesławska E., Satała G., Nitek W., Partyka A., Siwek A., Jankowska A., Wesołowska A. (2018). Computer-aided insights into receptor-ligand interaction for novel 5-arylhydantoin derivatives as serotonin 5-HT 7 receptor agents with antidepressant activity. Eur. J. Med. Chem..

[27] Gaulton A., Bellis L.J., Bento A.P., Chambers J., Davies M., Hersey A., Light Y., McGlinchey S., Michalovich D., Al-Lazikani B. (2012). ChEMBL: A large-scale bioactivity database for drug discovery. Nucleic Acids Res..

[28] Bajusz D., Rácz A., Héberger K. (2015). Why is Tanimoto index an appropriate choice for fingerprint-based similarity calculations?. J. Cheminform..

[29] Pándy-Szekeres G., Munk C., Tsonkov T.M., Mordalski S., Harpsøe K., Hauser A.S., Bojarski A.J., Gloriam D.E. (2018). GPCRdb in 2018: Adding GPCR structure models and ligands. Nucleic Acids Res..

[30] Desaphy J., Raimbaud E., Ducrot P., Rognan D. (2013). Encoding protein-ligand interaction patterns in fingerprints and graphs. J. Chem. Inf. Model..

[31] Fisher R.A. (1915). Frequency Distribution of the Values of the Correlation Coefficient in Samples from an Indefinitely Large Population. Biometrika.

[32] Handzlik J., Szymańska E., Alibert S., Chevalier J., Otrȩbska E., Pȩkala E., Pagès J.M., Kieć-Kononowicz K. (2013). Search for new tools to combat Gram-negative resistant bacteria among amine derivatives of 5-arylidenehydantoin. Bioorganic Med. Chem..

[33] Marton J., Enisz J., Hosztafi S., Tímár T. (1993). Preparation and Fungicidal Activity of 5-Substituted Hydantoins and Their 2-Thio Analogs. J. Agric. Food Chem..

[34] Handzlik J., Kieć-Kononowicz K., Dela A., Otrębska E., Kaleta M. (2015). Novel N3-aminoalkyl Derivatives of 5-arylidenehydantoin; Pharmaceutical Composition Containing the Above and Application thereof.

[35] Cheng Y., Prusoff W.H. (1973). Relationship between the inhibition constant (K1) and the concentration of inhibitor which causes 50 per cent inhibition (I50) of an enzymatic reaction. Biochem. Pharmacol..

[36] Rogers D., Hahn M. (2010). Extended-connectivity fingerprints. J. Chem. Inf. Model..

[37] JChem 18.3.0, 2018, ChemAxon. http://www.chemaxon.com.

[38] (2020). LigPrep, Schrödinger Release 2020-1, LLC, New York, NY, USA. https://www.schrodinger.com/products/ligprep.

[39] (2020). Glide, Schrödinger Release 2020-1, LLC, New York, NY, USA. https://www.schrodinger.com/products/glide.

[40] (2020). Schrödinger Release 2020-1: Desmond Molecular Dynamics System, D.E. Shaw Research, New York, NY, 2020. Maestro-Desmond Interoperability Tools.

[41] Jorgensen W.L., Chandrasekhar J., Madura J.D., Impey R.W., Klein M.L. (1983). Comparison of simple potential functions for simulating liquid water. J. Chem. Phys..

