# Supplementary material for: The Structural Determinants for α1-Adrenergic/Serotonin Receptors Activity among Phenylpiperazine-Hydantoin Derivatives
Source: Molecules. 2021 Nov 20;26(22):7025. doi: 10.3390/molecules26227025 (PMC8623851; doi:10.3390/molecules26227025)

Supporting Information

The structural determinants for α_1-adrenergic/serotonin receptors activity among phenylpiperazine-hydantoin derivatives.

Katarzyna Kucwaj-Brysz[a], Anna Dela[a], , Marek Bednarski, Sabina Podlewska[a,b], Agata Siwek[d], Grzegorz Satała[b], Kinga Czarnota[a] Jadwiga Handzlik[a], Katarzyna Kieć-Kononowicz[a*]

[1] Faculty of Pharmacy, Department of Technology and Biotechnology of Drugs, Jagiellonian University Medical College, 9 Medyczna Street, 30-688 Kraków, Poland
[2] Maj Institute of Pharmacology, Polish Academy of Sciences, 12 Smętna Street, 31-343 Kraków, Poland
[3] Department of Pharmacodynamics, Jagiellonian University Medical College, Medyczna 9 Street, 30-688 Kraków, Poland
[4] Department of Pharmacobiology, Jagiellonian University Medical College, Medyczna 9 Street, 30-688 Kraków, Poland
* Correspondence: katarzyna.kucwaj@uj.edu.pl; katarzyna.kiec-kononowicz@uj.edu.pl
† Equal contribution

Table of contents

1. ^{1}H and ^{13}C NMR spectra for final compounds

Figure S1. *(Z)-3-(2-hydroxy-3-(4-(2-methoxyphenyl)piperazin-1-yl)propyl)-5-(3-methoxybenzylidene)imidazolidine-2,4-dione* (**4**)

^{1}H NMR (500 MHz, DMSO-d$_6$)

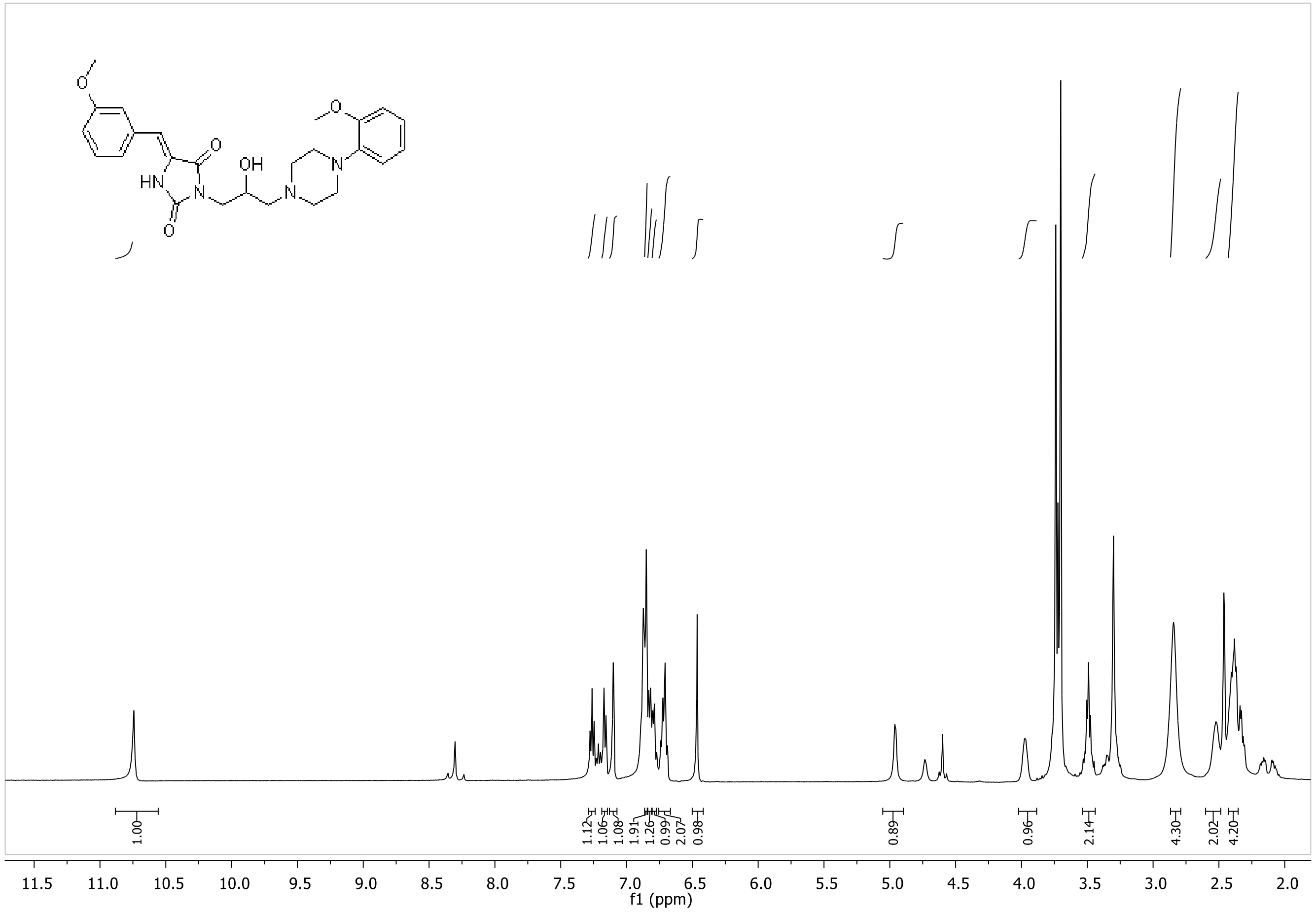

Figure S2. AD09 *(Z)-3-(3-(4-(2-ethoxyphenyl)piperazin-1-yl)-2-hydroxypropyl)-5-(3-methoxybenzylidene)imidazolidine-2,4-dione* (**5**)

^{1}H NMR (500 MHz, DMSO-d_6)

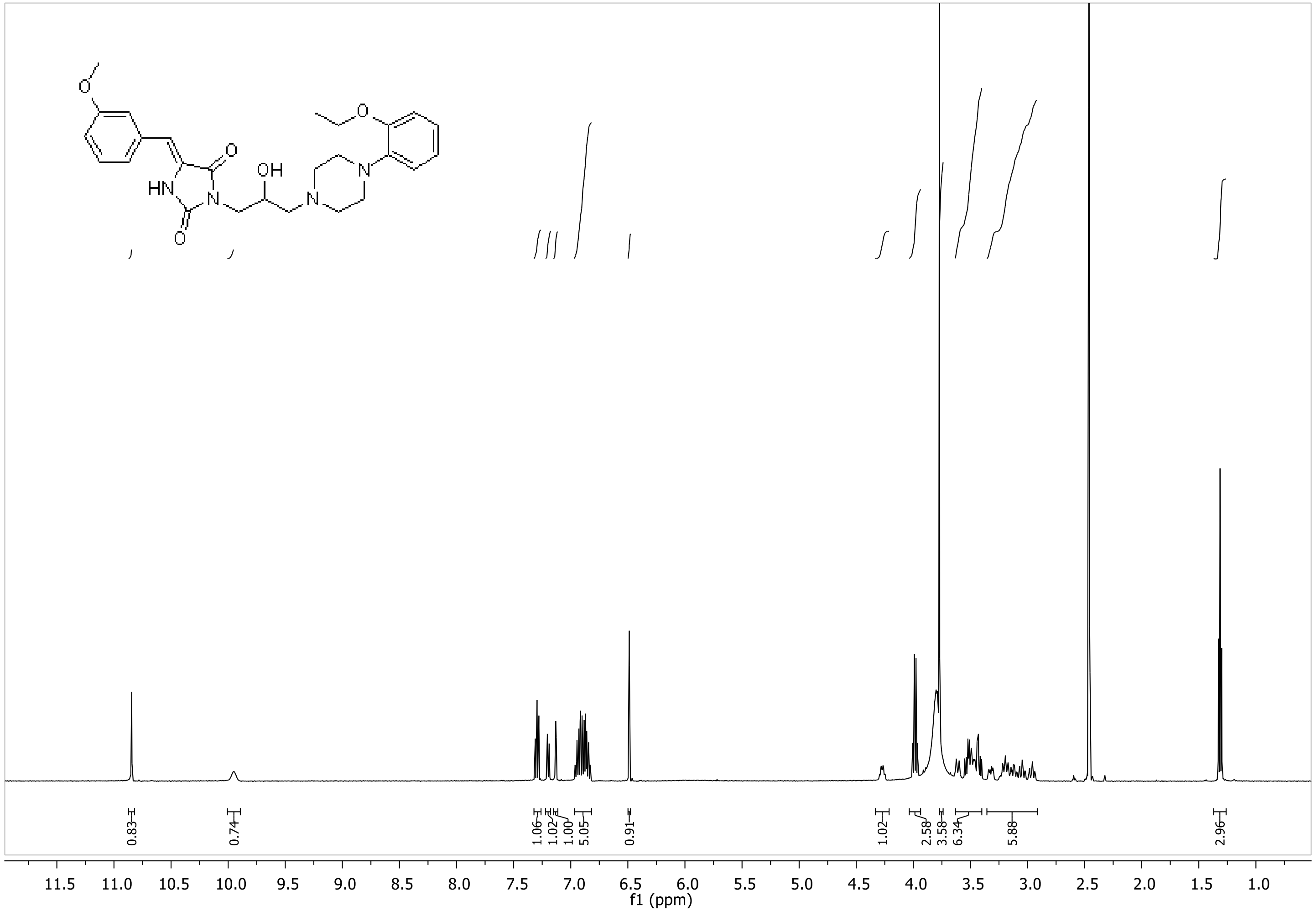

Figure S3. AD15 *(Z)-3-(2-hydroxy-3-(4-(2-methoxyphenyl)piperazin-1-yl)propyl)-5-(4-methoxybenzylidene)imidazolidine-2,4-dione* (**6**)

^{1}H NMR (500 MHz, DMSO-d_6)

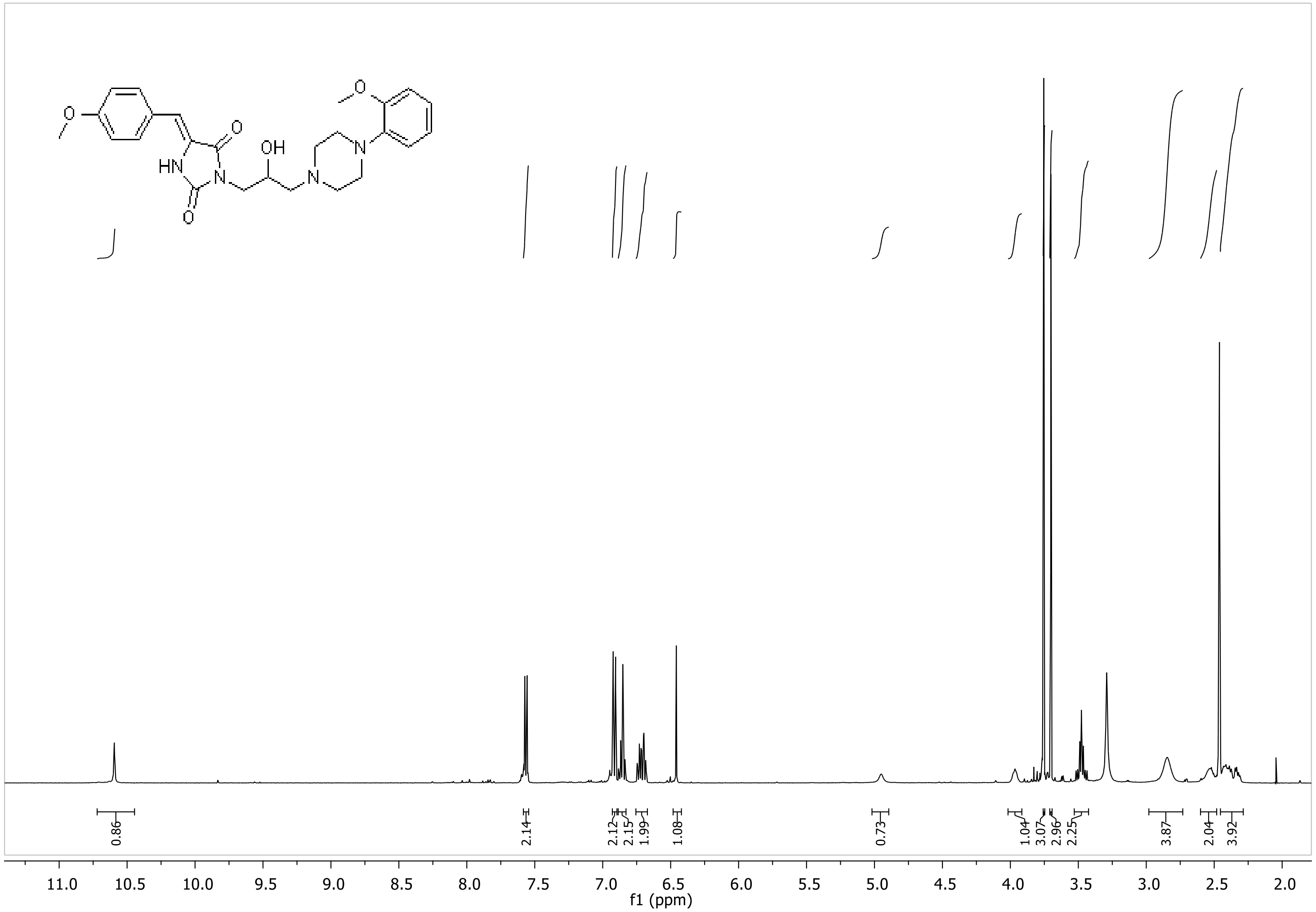

Figure S4.. AD13 *(Z)-3-(3-(4-(2-ethoxyphenyl)piperazin-1-yl)-2-hydroxypropyl)-5-(4-methoxybenzylidene)imidazolidine-2,4-dione* (**7**)

^{1}H NMR (500 MHz, DMSO-d$_6$)

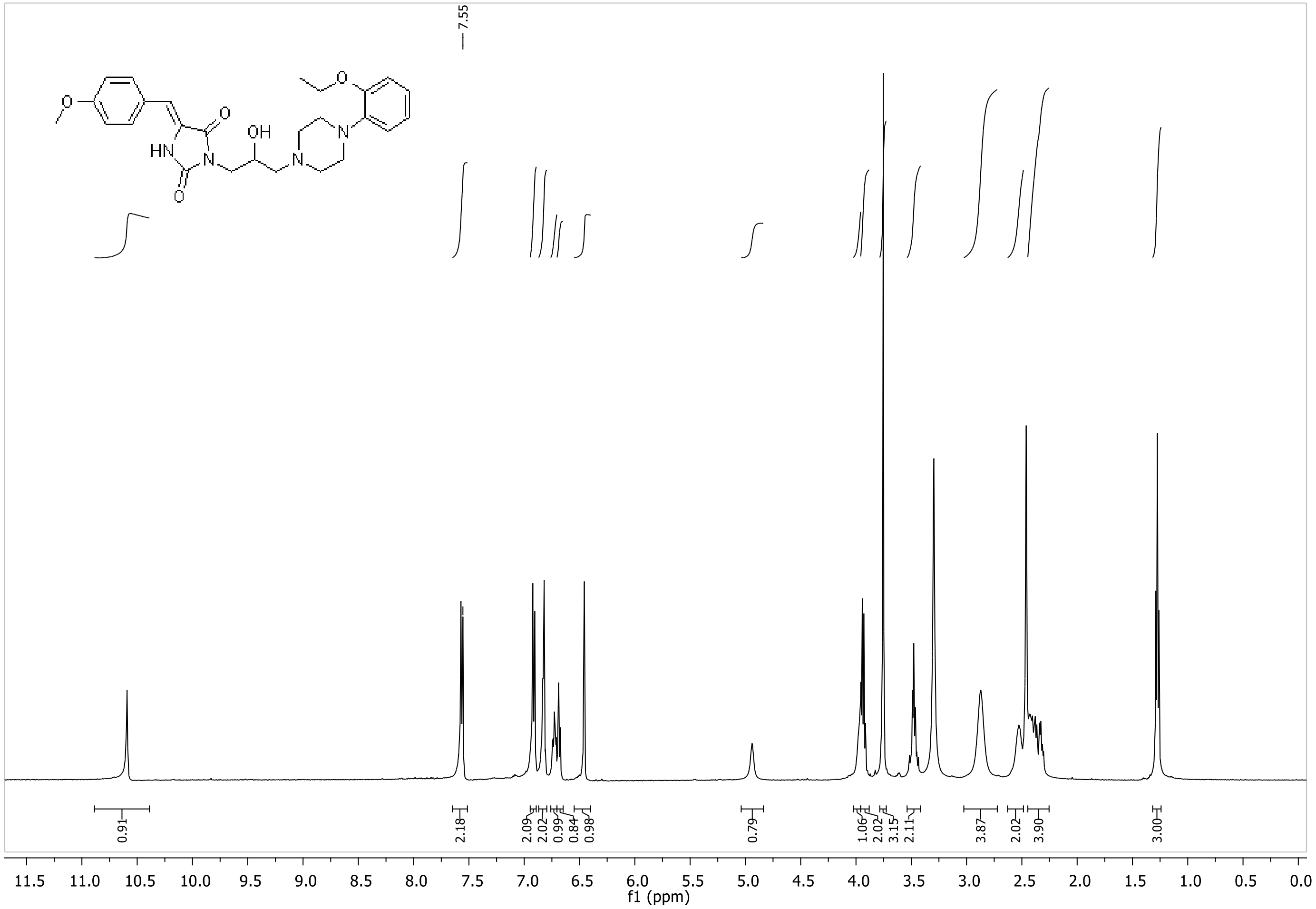

^{13}C NMR (125 MHz, DMSO-d_6)

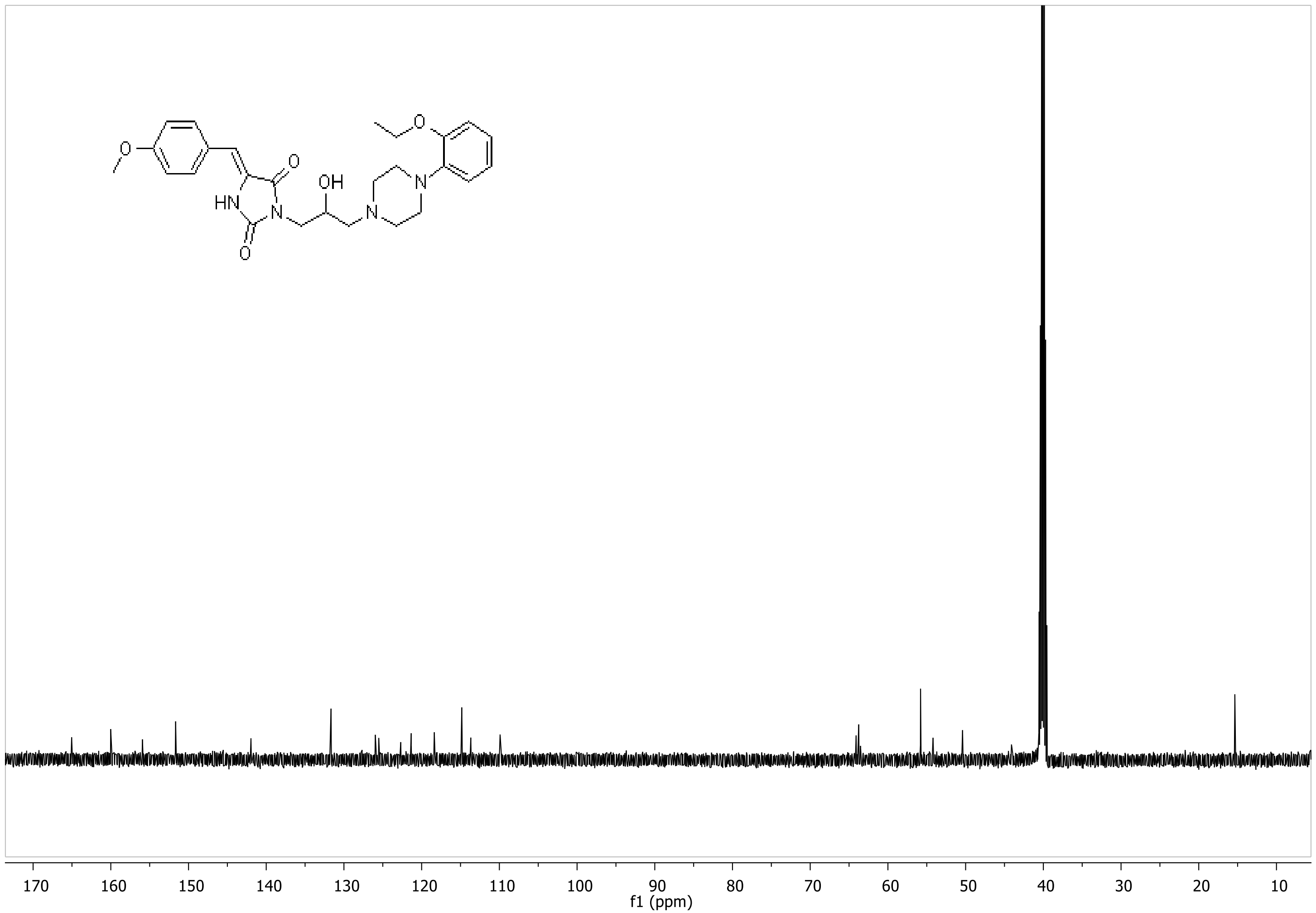

Figure S5. AD38 *(Z)-3-(3-(4-(2-fluorophenyl)piperazin-1-yl)-2-hydroxypropyl)-5-(4-methoxybenzylidene)imidazolidine-2,4-dione* (**8**)

^{1}H NMR (500 MHz, DMSO-d_6)

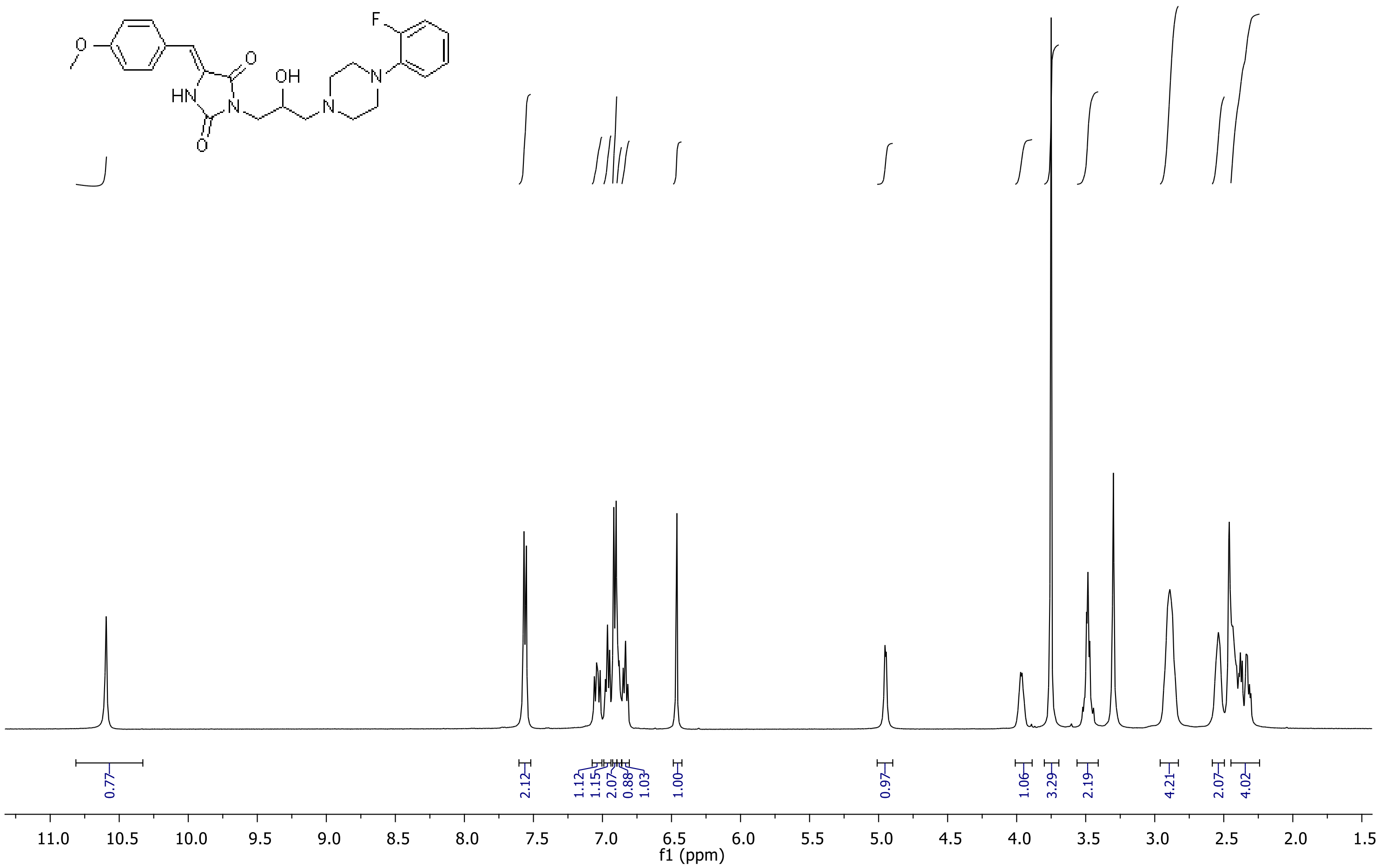

^{13}C NMR (125 MHz, DMSO-d_6)

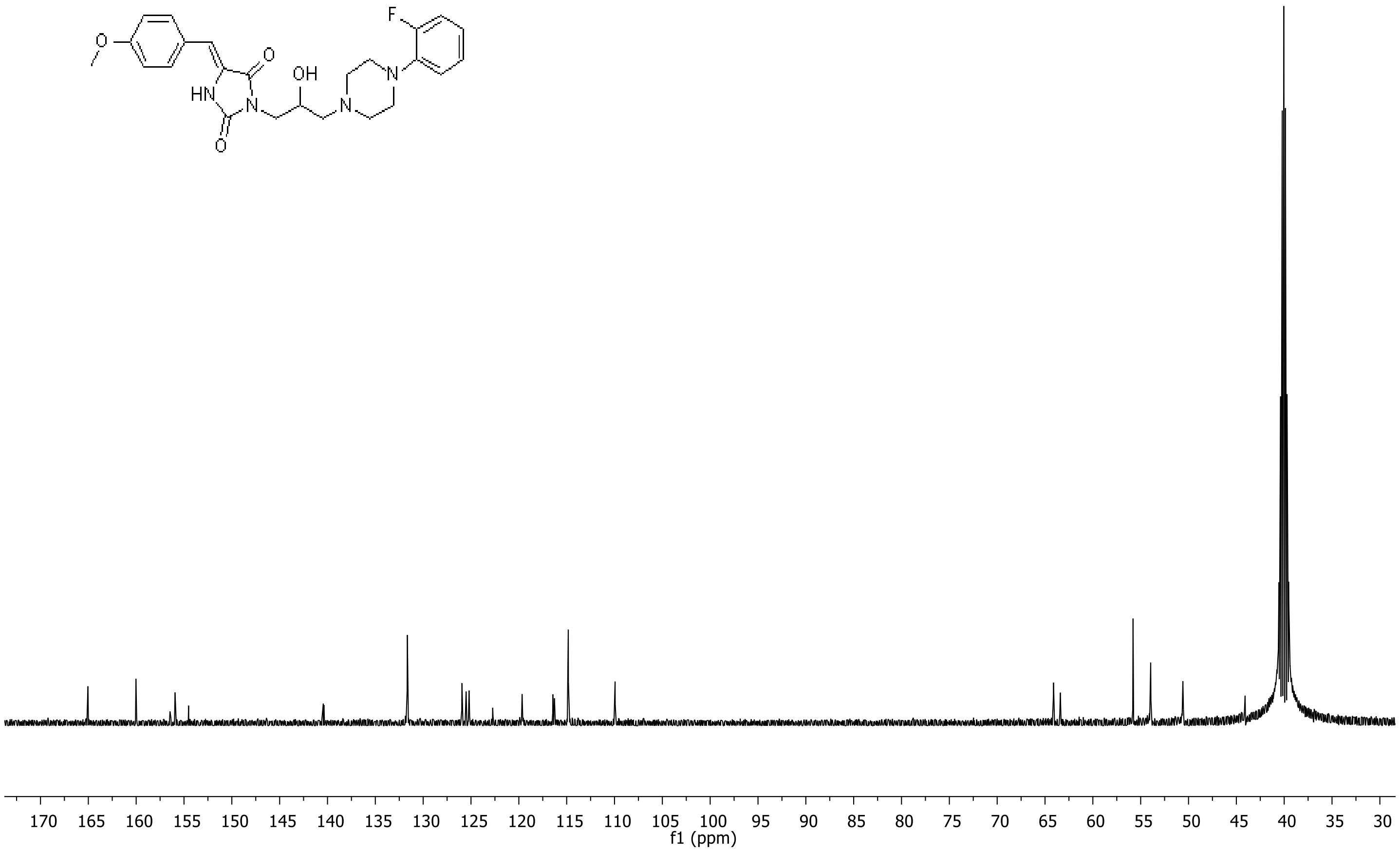

Figure S6. /AD34 *(Z)-3-(3-(4-(2-ethoxyphenyl)piperazin-1-yl)-2-hydroxypropyl)-5-(3,4,5-trimethoxybenzylidene)imidazolidine-2,4-dione* (**9**)

^{1}H NMR (500 MHz, DMSO-d_6)

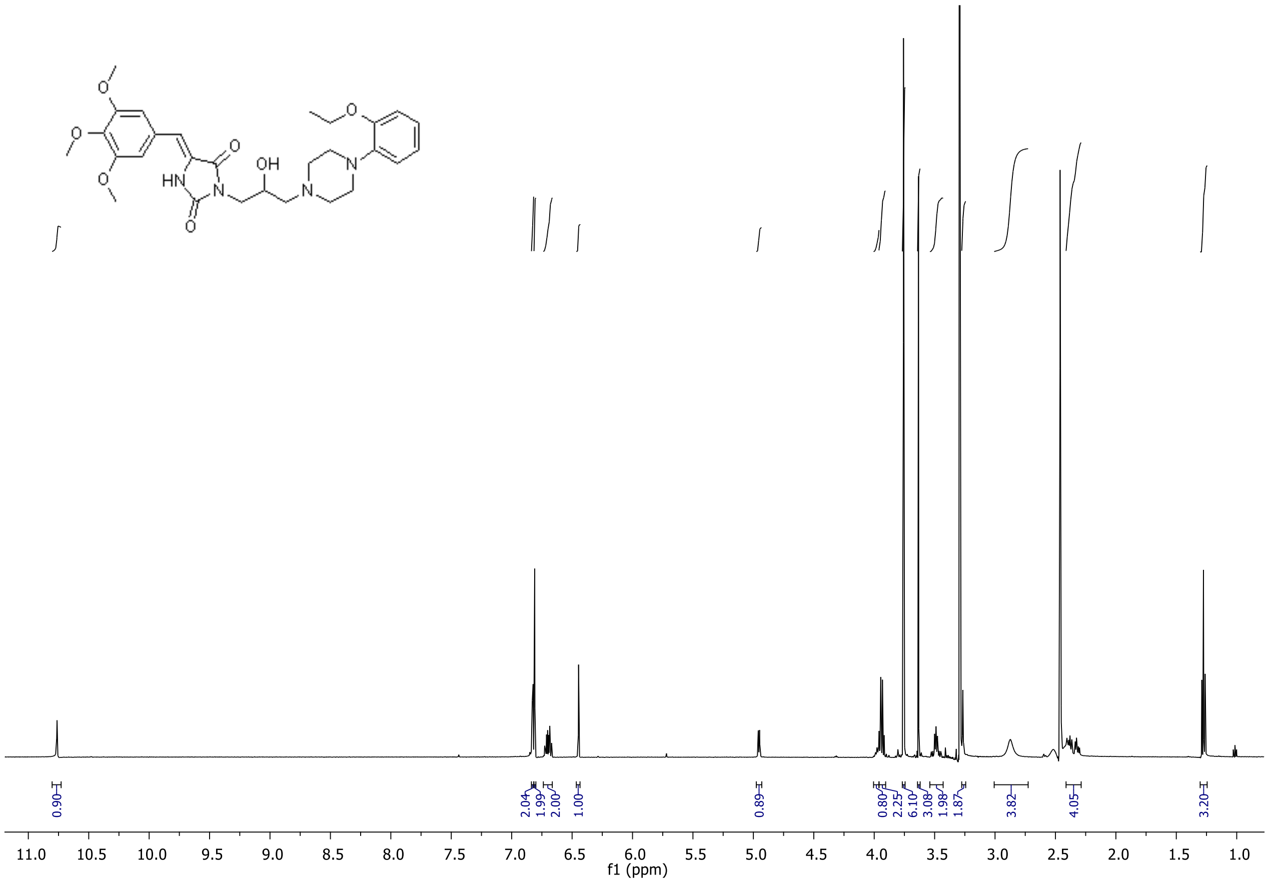

Figure S7. AD42 *(Z)-3-(3-(4-(2-chlorophenyl)piperazin-1-yl)propyl)-5-(3-methoxybenzylidene)imidazolidine-2,4-dione* (**11**)

^{1}H NMR (500 MHz, DMSO-d_6)

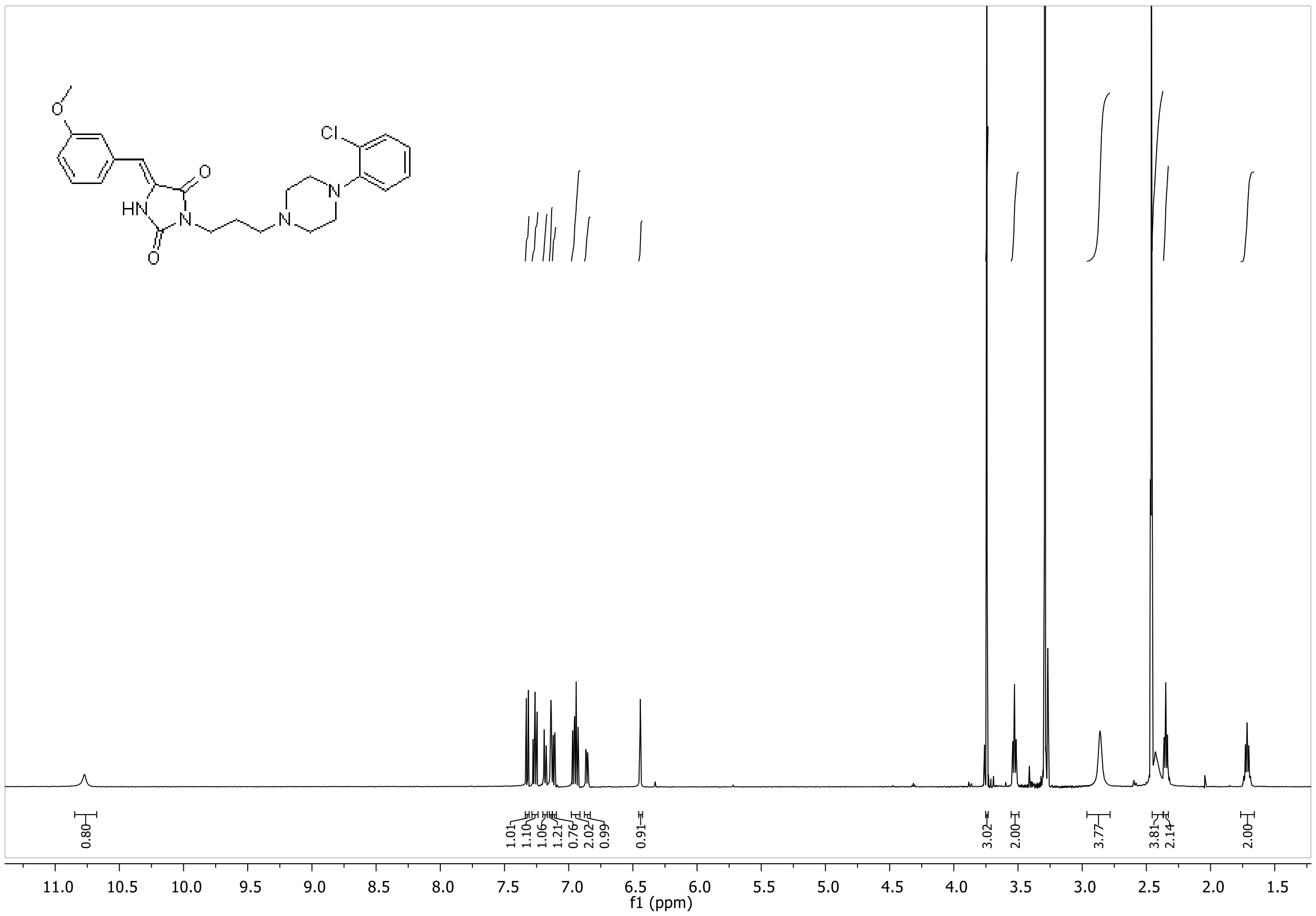

Figure S8. AD26 *(Z)-5-(4-methoxybenzylidene)-3-(3-(4-(2-methoxyphenyl)piperazin-1-yl)propyl)imidazolidine-2,4-dione* (**12**)

1H NMR (500 MHz, DMSO-d_6)

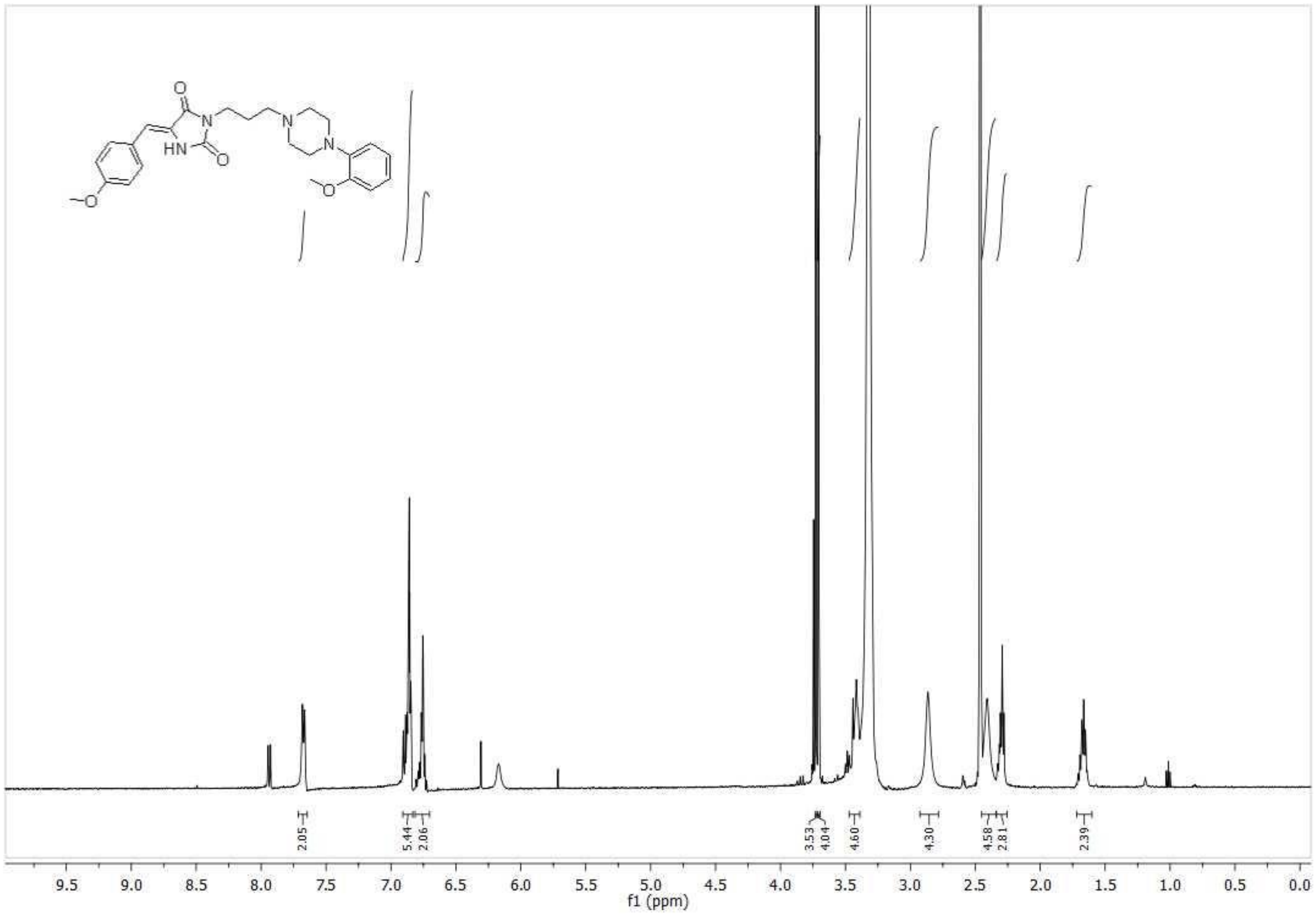

Figure S9. AD30 *(Z)-3-(3-(4-(2-ethoxyphenyl)piperazin-1-yl)propyl)-5-(4-methoxybenzylidene)imidazolidine-2,4-dione* (**13**)

^{1}H NMR (500 MHz, DMSO-d_6)

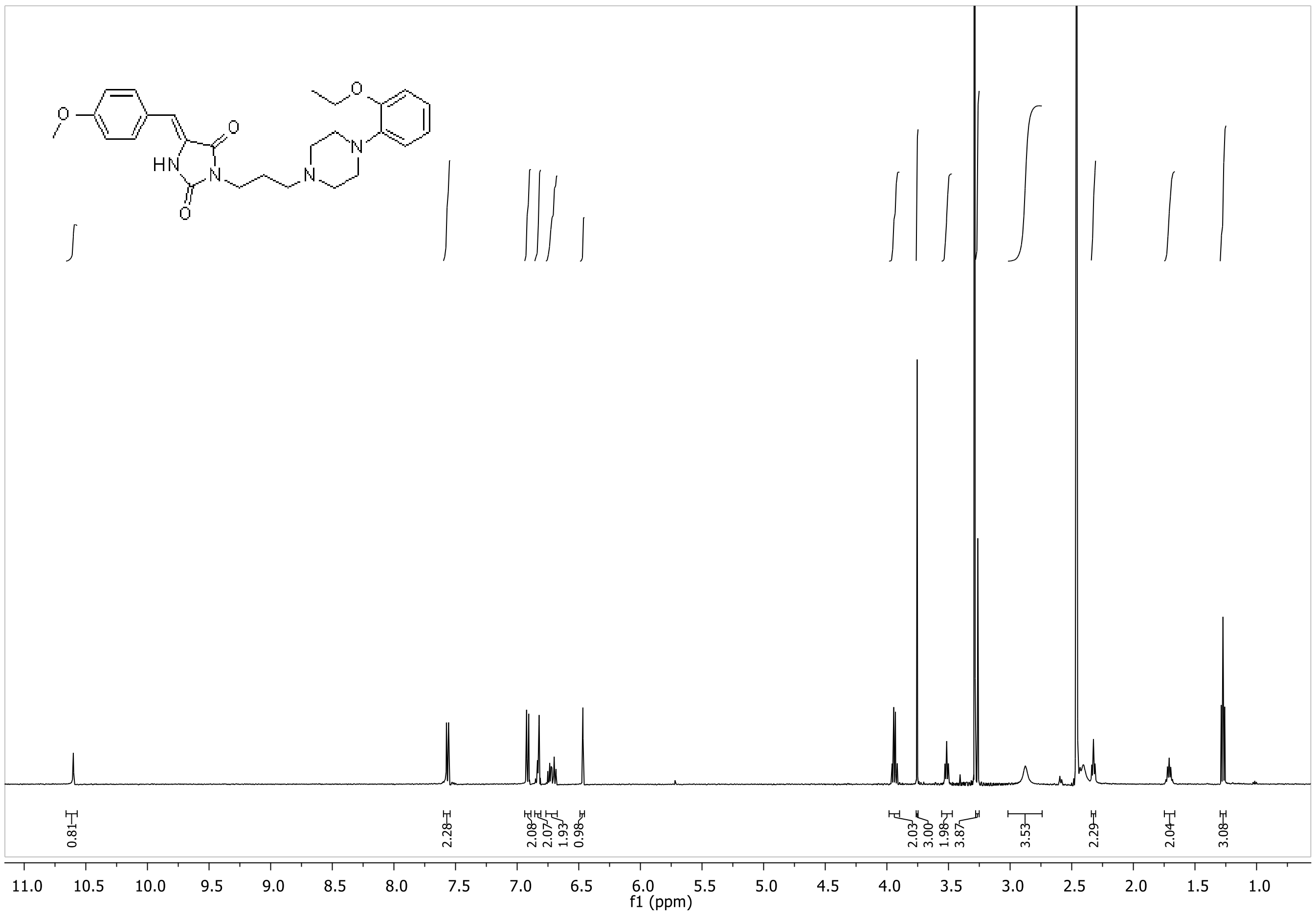

Figure S10. AD28 *(Z)-3-(3-(4-(2-methoxyphenyl)piperazin-1-yl)propyl)-5-(3,4,5-trimethoxybenzylidene)imidazolidine-2,4-dione* (**15**)

^{1}H NMR (500 MHz, DMSO-d_6)

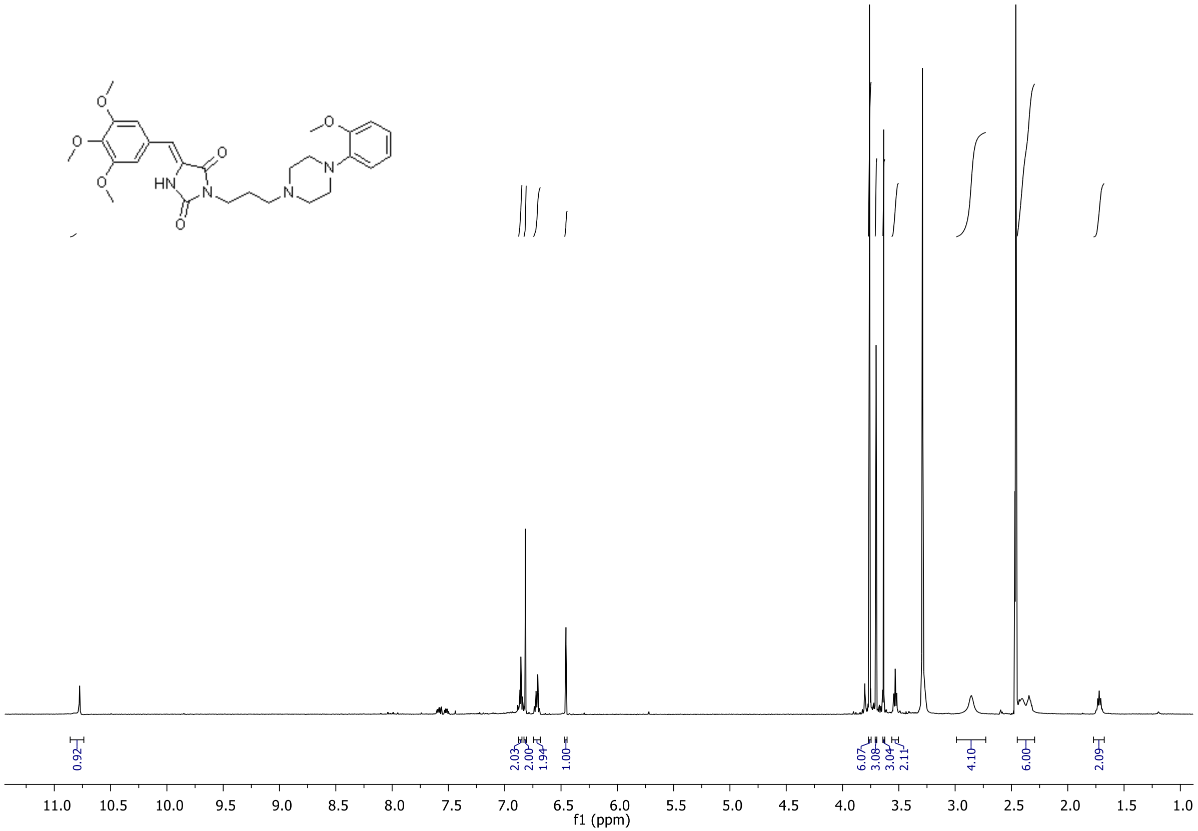

Figure S11. AD33 *(Z)-3-(3-(4-(2-ethoxyphenyl)piperazin-1-yl)propyl)-5-(3,4,5-trimethoxybenzylidene)imidazolidine-2,4-dione* (**16**)

^{1}H NMR (500 MHz, DMSO-d$_6$)

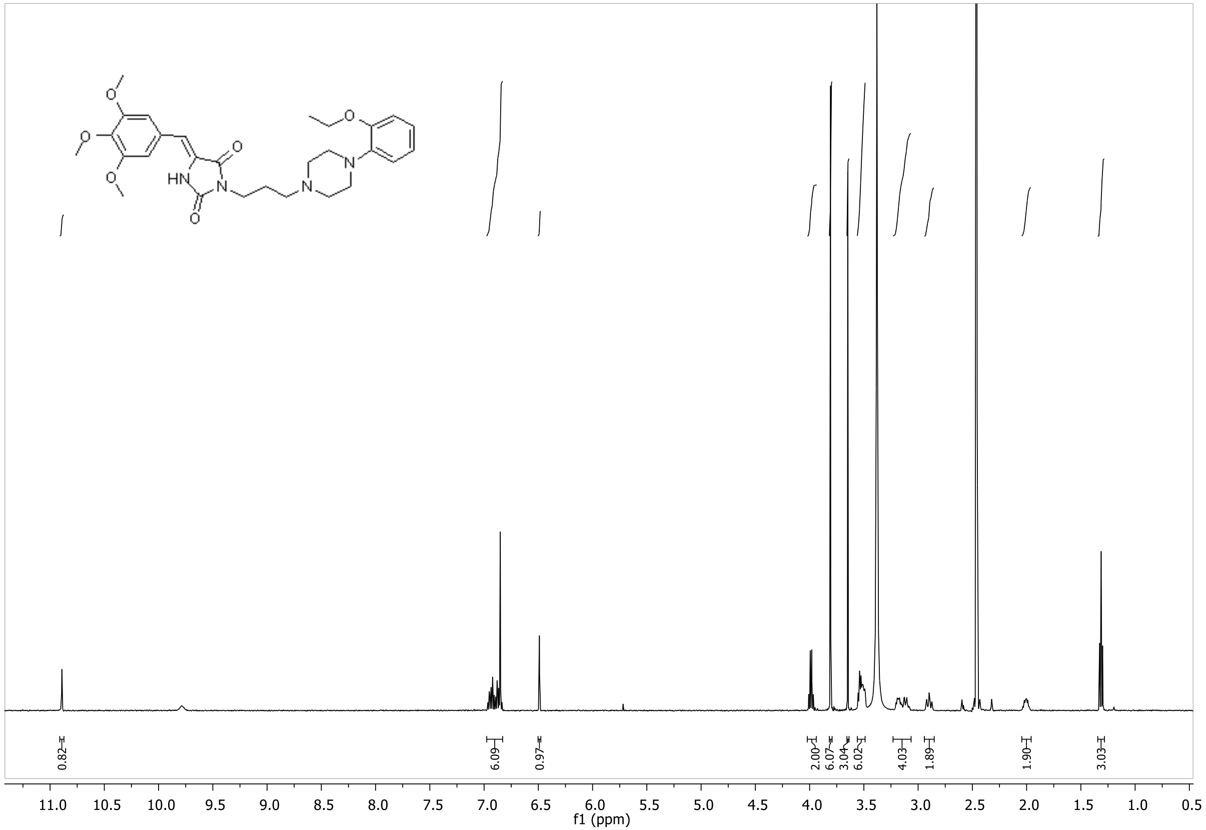

Supplement: Supplementary file 1 [file molecules-26-07025-s001.zip › molecules-1460118-supplementary.pdf]
